# Prospects on the nano-plastic particles internalization and induction of cellular response in human keratinocytes

**DOI:** 10.1186/s12989-021-00428-9

**Published:** 2021-09-08

**Authors:** Ponnusamy Manogaran Gopinath, Krishna Sundar Twayana, Palaniyandi Ravanan, John Thomas, Amitava Mukherjee, David F. Jenkins, Natarajan Chandrasekaran

**Affiliations:** 1 Centre for Nanobiotechnology, Vellore Institute of Technology (VIT), Tamil Nadu, Vellore, 632 014 India; 2 Apoptosis and Cell Survival Research Lab, Department of Biosciences, School of Biosciences and Technology, VIT University, Vellore, Tamil Nadu 632 014 India; 3 Department of Microbiology, School of Life Sciences, Central University of Tamil Nadu, Thiruvarur, Tamil Nadu 610 104 India; 4 Faculty of Science and Environment, Plymouth University, Plymouth, PL4 8AA UK

**Keywords:** Nano-plastics, Keratinocytes, Human keratin, Protein-corona, Macropinocytosis, Oxidative stress, Autophagy, Senescence

## Abstract

**Background:**

Today, cosmetic products are very popular with both men and women to improve their appearance and increase their social acceptability.

**Results:**

In this study, nano-sized (30–300 nm) plastic particles were isolated from the commercial face-scrubs and treated on the human keratinocytes. The observed adherence of polyethylene nano-plastics (PENPs), polystyrene NPs (PSNPs), and face-scrubs isolated nano-plastics (NPs) on the keratin layer reveals a significant attachment of NPs from the cosmetics that are applied on the skin for a short duration. This attachment property could facilitate further adherence of protein molecules on NPs and the protein-corona formation. The protein-corona mimics protein aggregates, thereby triggers macropinocytosis, followed by the macropinolysosomal process in the cell. These internalized NPs induced the concentration-dependent cytotoxic, cytostatic and cytoprotective activity in keratinocytes. Both single dose and chronic long-term exposure of lethal and sub-lethal concentrations of NPs resulted in oxidative stress-mediated down-regulation of cell growth and proliferation inhibition. Autophagic structures and premature aging were also observed using an electron microscopy and a senescence marker, respectively in the NPs internalized HaCaT cells incubated in a fresh, NPs-free medium.

**Conclusion:**

Though 2D culture models have many limitations, it produces significant conceptual advancements. This work provides an insight into the NPs concentration-dependent regulatory, cytoprotective, and cytotoxic effects in HaCaT cells. However, 3D model studies are required to identify the detailed mechanisms of NPs toxicity and cytoprotective events in cells at the molecular level.

**Graphic abstract:**

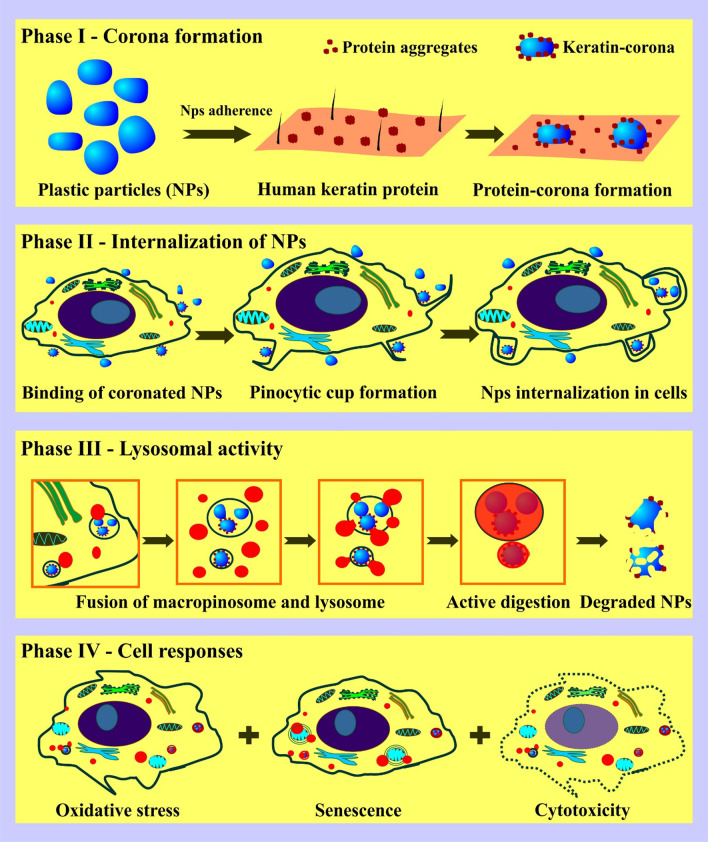

**Supplementary Information:**

The online version contains supplementary material available at 10.1186/s12989-021-00428-9.

## Background

Fashion is an aesthetic declaration that strengthens a person's appearance and provides better social visibility and acceptibilty [1, 2]. Cosmetics are not a modern invention. Both men and women in ancient Egypt (4000 BC), India (2500 BC), and China (3000 BC) have used cosmetics to alter their appearance or as a sign of nobility or religion [3, 4]. Today, people of all age groups use cosmetics as a beautifying agent (makeup and lipsticks), cleansing agent (soaps, shower gels, shampoos, face scrubs, and toothpaste), odour-fighting agents (perfumes, deodorants, and anti-perspirants), and protective agents (sunscreens and toothpastes for sensitive teeth) [5–7]. Earlier, the cosmetics and personal care products were composed of natural exfoliants such as pumice, aniseed, coconut, almonds, oat flour, fruit stones, and husk of apricot, bamboo, pecan nut, and walnut shell [5] besides the conventional ingredients, such as soap, surfactant, alcohol, oil, wax, water, and inorganic pigments. Recently, nano- and micro-sized plastic polymers such as polyethylene (PE), polypropylene, polystyrene (PS), and nylon have replaced the natural exfoliants, owing to their cost-effectiveness, functionality, versatility, durability, lightweight, and corrosion-resistant properties [5–10].

According to the European Chemical Agency (ECHA) and United Nations Environment Programme (UNEP) reports, more than 500 different types of nano- and micro-plastic ingredients, with a combined weight of more than 9300 tonnes are added to cosmetics and personal care products (CPCP) every year in European Union countries alone [7, 11]. Polyethylene (PE) polymer particles are commonly added in cosmetics [7] because of their abrasiveness, film-forming, and viscosity controlling capacity, followed by polypropylene (PP) and polystyrene (PS) particles. Until now, there is no specific data available on the range of sizes of plastic particles used in cosmetics. Several studies found particles around 5 µm to 2 mm-sized in major cosmetic brands. The size-frequency distribution data showed that most of the products consist of a wide range of plastic particles, whereas some products were the more homogenous with a large number of smaller particles [5, 9, 12]. Recently, Hernandez et al. [13] and our group [14, 15] have isolated sub 100 nm-sized nano-plastic (NPs) particles from facial scrubs. According to Hernandez et al., one gram of face scrub contains more than 300 billion nano-sized plastic particles equivalent to 300 µg [13]. It has been estimated that the PE microplastics (MPs) usage by the US population via CPCP is around 2.4 mg/person/day [16]. The PEMPs release from the facial scrub usage by the UK population ranges between 40.5 and 215 mg/person/day, suggesting the annual environmental release of ~ 86 tonnes of MPs from facial exfoliants alone [9].

Numerous studies have proved that CPCP is the primary contributor to aquatic plastic pollution [17] and have shown the adverse effects of microplastics on various marine and terrestrial organisms. Only a few studies have demonstrated the toxicity of environmentally isolated microplastics on human cells [15, 18], plant cells [15], and marine animals [19]. Most of the studies used commercial virgin-NPs or -MPs and showed altered growth and reproduction in rotifer, amphipod, and copepod [20–22]. Also, virgin-NPs/ MPs affected cellular function in blue mussels and sea bass [23, 24], reduced feeding activity in lugworm [25], activated fibrosis, congestion, and inflammatory infiltration in the earthworm [26], and triggered the immune response in mice [27]. Consequently, in the 2nd Environmental Conference, 2015, plastic particles were recognized and described as the second most poisonous agent that affects ecology and the environment [28]. This perception raised concerns about the direct exposure of plastic particles to human skin via cosmetics. Subsequently, it warranted an empirical investigation on the effect of nano-plastic on skin cells.

The skin is made up of epidermis, dermis, and subcutaneous layers and serves as a physical barrier against fluctuating environmental factors, including pollutants, toxic substances, ultraviolet and ionizing radiation. Additionally, the skin also performs several vital functions, including defensive, thermoregulatory, sensory, and excretory. The epidermis is the external layer composed of 90–95% keratinocytes and fewer melanocytes and Langerhans cells [29]. Direct application of NPs and MPs incorporated cosmetics on the skin could allow high penetration of NPs via percutaneous absorption [30]. But the adsorption and persistence of NPs on the skin and their effects on keratinocytes are understudied. Recently, several studies focused on the impacts of unintentionally exposed NPs via ingestion [31] or inhalation route [32]. But very few studies have demonstrated the penetration and accumulation tendency of polymeric NPs on inflamed skin [33, 34]. However, the effects of NPs found in cosmetics on human skin cells are not known. Hence, the present study intended to determine the entry of polyethylene NPs (PENPs), polystyrene NPs (PSNPs), and NPs isolated from the face scrubs (FS) and their effect on human keratinocytes.

## Results and discussion

### Isolation and determination of NPs from face scrubs

Face scrubs (FS) manufactured by two different industries were purchased from the local supermarket and used for NPs extraction by sequential filtration [13]. Since the brand names are not particularly relevant to the study, we presented the samples as FS-1 and FS-2. The FS-1 and FS-2 are used by men and women, respectively. Both products contain common ingredients such as antioxidants, preservatives, and fragrance enhancers. Notably, FS-2 comprises polyquaternium, a cosmetic ingredient designated for several cationic polymers with different properties. Regardless of the ingredient list, we have observed MPs and NPs in both products. The NPs particles isolated from FS-1 and FS-2 were termed as NPs-1 and NPs-2, respectively.

**Fig. 1:**
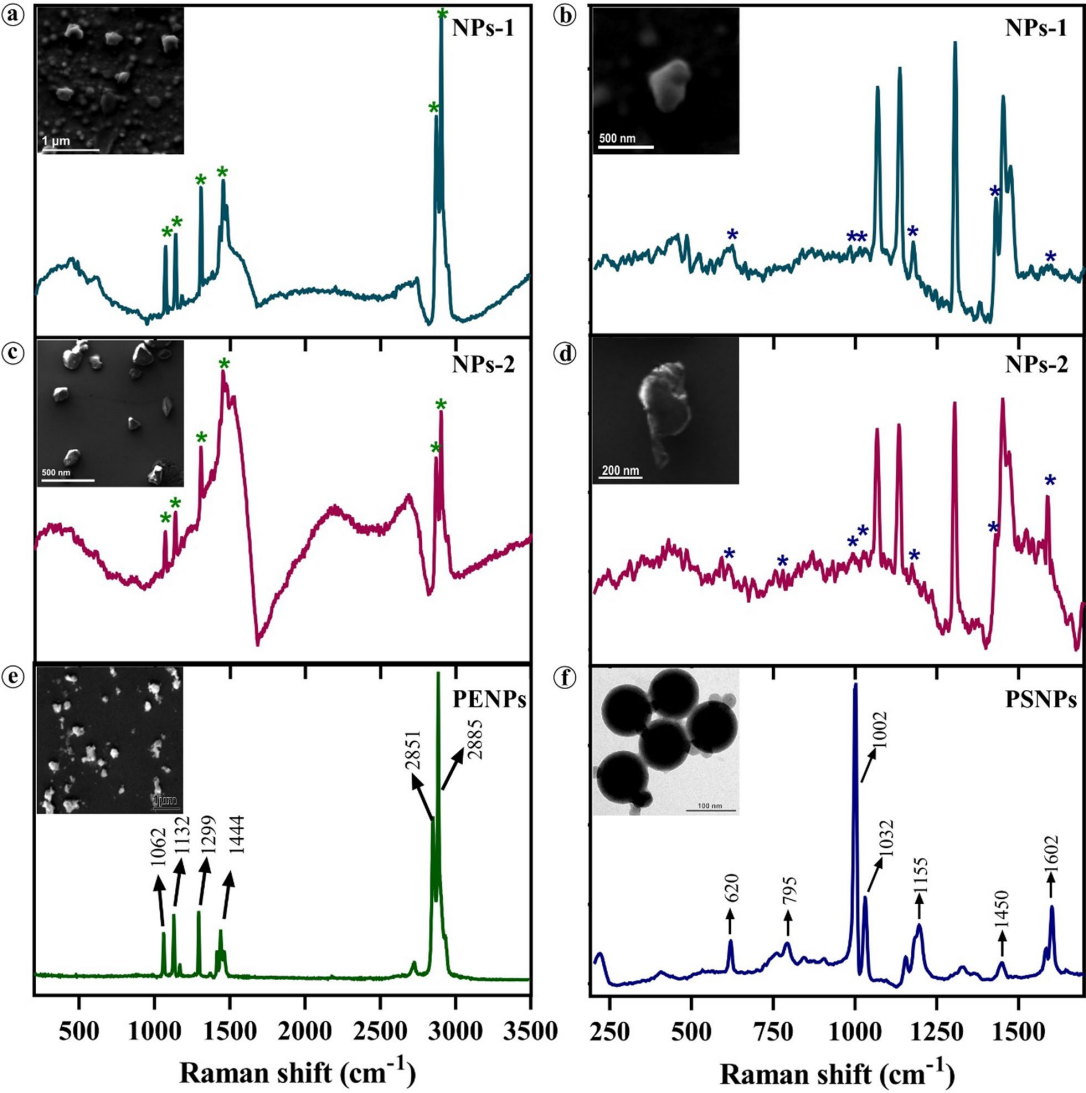
Characterization NPs isolated from cosmetics. **a**, **b** Raman spectrum of NPs-1 isolated from face scrub, Insert; HR-SEM micrograph of NPs-1. **c**, **d** Raman spectrum of NPs-2 isolated from face scrub, Insert; HR-SEM micrograph of NPs-2. **e** Standard Raman spectrum of Polyethylene NPs, Insert; HR-SEM micrograph of PENPs. **f** Standard Raman spectrum of Polystyrene NPs, Insert; HR-TEM micrograph of PSNPs. **a**, **c**, **e** Raman spectra collected in the 200–3500 cm^−1^ range and **b**, **d**, **f** Raman spectra collected in the 200–1700 cm^−1^ range

The NPs containing final filtrates obtained from the FS samples via subsequent filtration were examined under the high-resolution scanning electron microscope (HR-SEM) to determine their hydrodynamic size and shape. The electron micrographs showed that the NPs-1 isolated from FS-1 are mostly spherical with a smooth surface and few irregular aggregates (Fig. 1a insert), whereas NPs-2 isolated from the FS-2 are amorphous with sharp edges (Fig. 1c insert). The amorphous nature of NPs may have resulted from the cosmetic production steps such as homogenization, emulsifying, and heating. The size distribution analysis showed a broad size distribution range from 30 to 300 nm and 90 to 230 nm for NPs-1 and NPs-2, respectively. In NPs-1, most of the particles are 100 ± 20 nm in diameter. The slight increase in the NPs particle size (> 200 nm) observed could be due to particle agglomeration during the drying process. On the other hand, the PENPs (Fig. 1c insert) prepared from the subsequent breakdown of PE pellets and the commercially procured virgin-PSNPs (Fig. 1d insert) showed irregular particles of about 400 nm or less in diameter and spherical particles of 100 nm in diameter, respectively. More details on the particle size distribution and NPs concentration in FS samples are available in the Additional file 1.

The dried NPs-1, NPs-2, PENPs, and PSNPs were analysed using Fourier Transform Raman Spectroscopy (FT-Raman). The Raman spectra of NPs-1 (Fig. 1a) and NPs-2 (Fig. 1c) showed a close correlation with the characteristic Raman bands of standard PENPs (Fig. 1e). The bands at 1062, 1132, 1299, and 1444 cm^−1^ are mainly due to the C–C stretching, CH_2_ twisting, and CH_2_ bending vibrations of PE, and the strong bands at 2851 and 2885 cm^−1^ are due to the CH_2_ asymmetric and symmetric stretching vibrations of PE [35, 36]. As mentioned above, PE is the most prominently used polymer (about 93%) in cosmetics; hence it could be the highest polymer fraction in the isolated NPs-1 and NPs-2. As a result, the PE Raman peaks might have surpassed the Raman shift of PS and other plastic polymers [7]. On closer examination, the Raman spectra of NPs-1 (Fig. 1b) and NPs-2 (Fig. 1d) showed the characteristic bands at 620, 795, 1002, 1032, 1155, 1450, and 1602 cm^−1^ corresponding to ring deformation, C–H out of plane deformation, ring breathing, C–H in-plane deformation, C–C stretch, CH_2_ scissoring, C=C stretch, and the ring-skeletal stretch of PSNPs (Fig. 1f), respectively. This observation indicates that both FS contains different types of nano-sized plastic particles, mainly polyethylene and polystyrene polymers.

### Dispersion and stability of NPs in the aqueous medium

The hydrodynamic size of PENPs, PSNPs, NPs-1, and NPs-2 in deionized water, keratin solution (0.2%), and Dulbecco's Modified Eagle's culture medium (DMEM) were measured using dynamic light scattering (DLS). During this experiment, the samples were vortexed vigorously in their respective solutions for 10 min and then incubated for 24 h at room temperature, or in cell culture conditions (at 37 °C with 5% CO_2_ and 95% humidity). The initial DLS measurements showed moderate stability (ζ ≥ −30 mV) of PENPS and PSNPs and an optimal dispersity and stability (ζ ≥ −40 mV) of NPs-1 and NPs-2 in deionized water (Table S1). But, over a 24 h period, the PENPs showed greater instability with a Z-average of 1409 ± 520 nm and PDI of 9.106, followed by a drastic decrease in the zeta potential value (− 9 ± 2.6 mV) in deionized water. On the other hand, all the NPs showed significant stability in the keratin solution. In DMEM, the average particle size of NPs-1 and NPs-2 increased 3 to fourfold compared to when in deionized water (Additional file 1: Table S1). The observed size increase in the NPs under the cell culture medium could be due to the aggregation of particles (Additional file 1: Figure S3d) via non-specific protein–protein attraction and the bridging effect between proteins and other biomolecules, as described in our previous study [15]. The above results indicate that the adsorption of biomolecules on the plastic particles plays a significant role in NPs aggregation and stability.

### Adherence of NPs particles on keratin coated glass slides

**Fig. 2:**
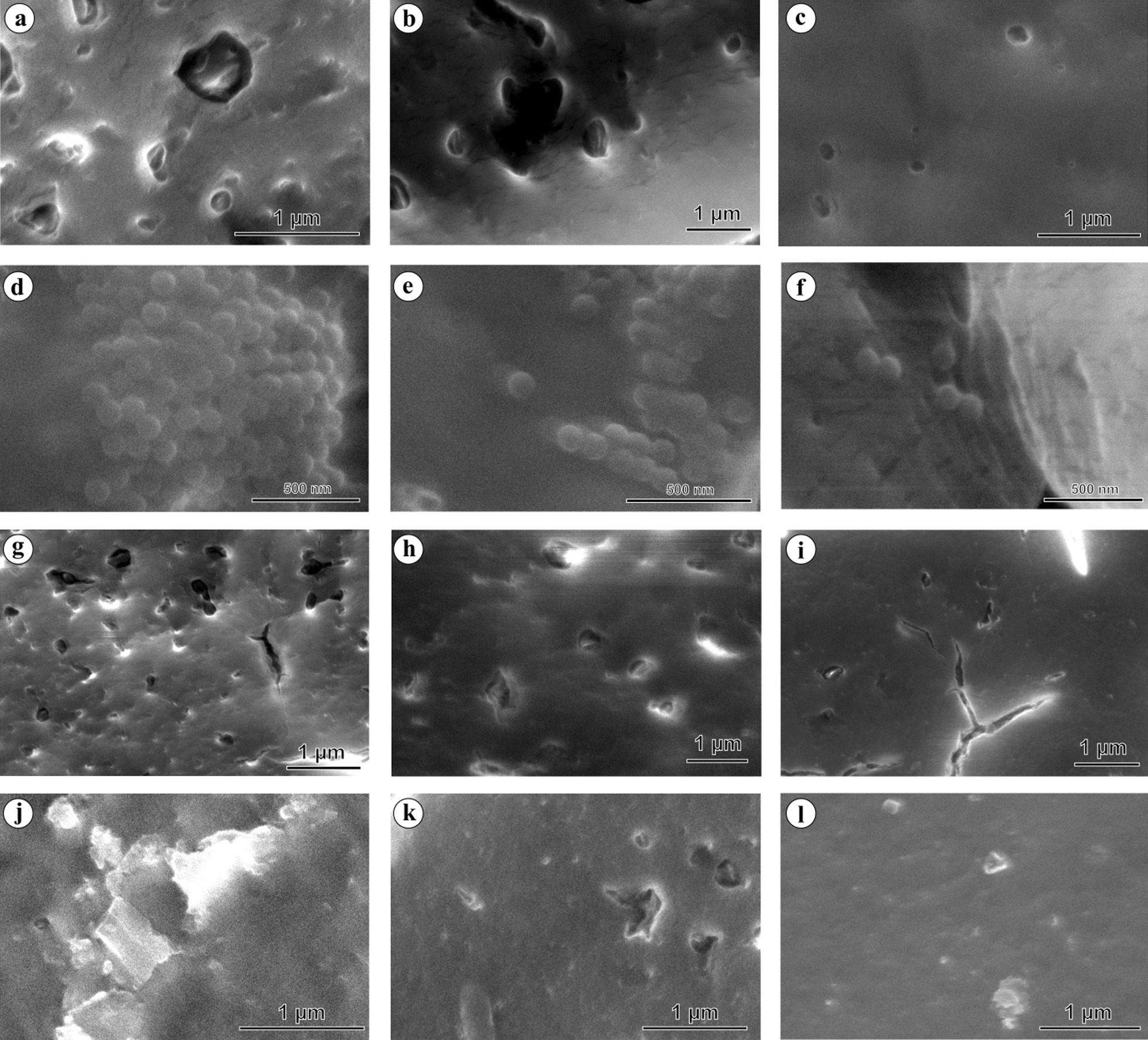
FE-SEM micrograph of nano-plastic particles adhered to the keratin coated glass slides (1 cm^2^). **a**, **b**, **c** PENPs, **d**, **e**, **f** PSNPs, **g**, **h**, **i** NPs-1, and **j**, **k**, **l** NPs-2. Electron micrographs of NPs exposed but unwashed glass slides (**a**, **d**, **g**, **j**), NPs exposed glass slides subjected to single wash (**b**, **e**, **h**, **k**), and NPs exposed glass slides subjected to two washes (8–10 dipping cycle per wash) (**c**, **f**, **i**, **l**)

It is a well-known phenomenon that NP/MP particles rapidly adhere to the biological substrates such as protein layers via non-specific attractive forces such as; Van der Waals forces, surface electric polarity alteration through a strong hydrophobic bond, dipole bonding by hydrogen bond via –OH, =O, –NH, =NH, ≡N groups, or spontaneous adsorption depending on the amino acid content [15]. The binding stability of NPs may differ depending on the attraction factors and contact/interaction time. To show the rapid attachment of NPs on the skin surface, we have exposed the keratin-coated glass slides to NPs solution (100 µg/mL) for 2–3 min and rinsed twice in ultra-pure water, and then observed under the electron microscope. Since this study uses NPs isolated from face scrubs, we have restricted the contact time for a maximum of 3 min and washed twice with deionized water. The field-emission scanning electron micrographs (FE-SEM) of keratin-coated glass slides showed a significant attachment of PENPs, PSNPs, NPs-1, and NPs-2 within 3 min of exposure (Fig. 2). Although washing removed most of the particles, a significant number of particles remained attached on the keratin-coated glass surface, even after two washes (8–10 dipping /wash) (Fig. 2c, f, i, and l). The NPs removal from the keratin layer during washing could be due to the weak adherence of NPs in the given contact time. However, the attached NPs on the protein layer, even after consecutive washes, revealed that washing does not remove all the particles adhered to the skin surface. Doge et al. [30] showed that the penetration of 20 and 200 nm-sized PSNPs into the stratum corneum was via skin furrows, lipid channels, and vellus hair follicles, and accumulation onto the viable epidermis just beneath the stratum corneum, as well as within the epidermis cells. Other studies have also reported that the stratum corneum could act as a long-term reservoir for the penetrated polymer particles and facilitate the NPs translocation into viable tissues [37–39].

### Impact of NPs on keratinocytes viability

**Fig. 3:**
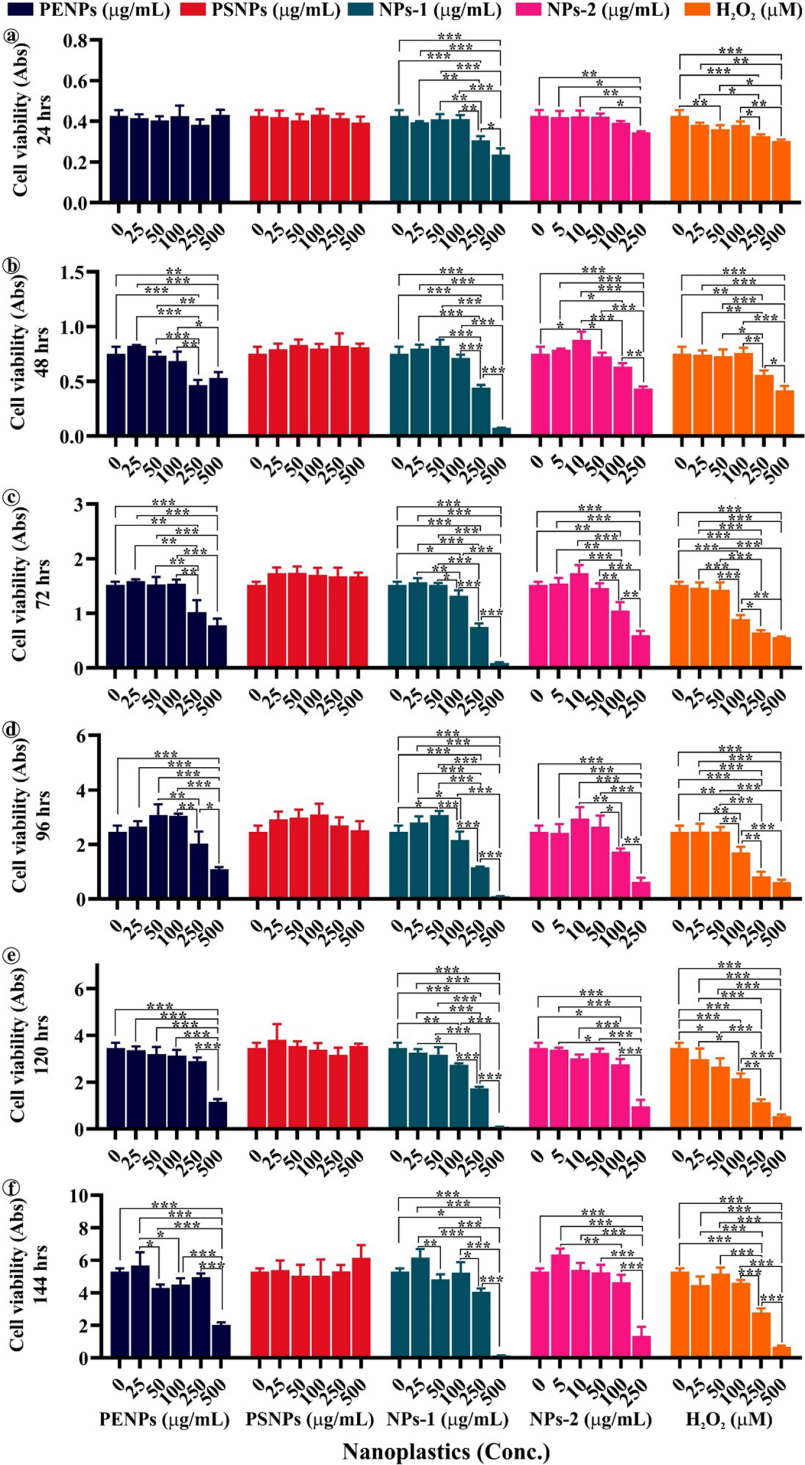
Dose-dependent cytotoxic effect of pristine and isolated NPs on the HaCaT cells at different time intervals. Treatment time interval **a** 24 h, **b** 48 h, **c** 72 h, **d** 96 h, **e** 120 h and **f** 144 h respectively for the PENPs, PSNPs, NPs-1, NPs-2 and H_2_O_2_. The data are presented as the means ± SEM of three independent experiments: *p ≤ 0.05, ** *p* ≤ 0.01, *** *p* ≤ 0.001 significantly different within the same NPs concentration

The constant use of cosmetics on the skin could lead to the long-term interaction of NPs with keratinocytes, thereby provoking physiological, biochemical, and pathological responses in cells. To observe the cytotoxic effect on the cell, we have exposed the keratinocytes to different concentrations of PENPs, PSNPs, NPs-1, and NPs-2 for six consecutive days. The MTT assay showed a gradual reduction in cell viability at high concentrations of PENPs, NPs-1, and NPs-2 with treatment time (Fig. 3). On the contrary, there was little or no growth inhibition observed in PSNPs and low concentrations of PENPs, NPs-1, and NPs-2 treated cells. The pairwise comparison of cell viability between the NPs dose range and control showed a statistically significant difference (*p* ≤ 0.05), evidencing the NPs concentration and time dependent toxicity in cells. Herein, we have limited the NPs-2 concentration range to 250 µg/mL, because of the complete growth inhibition of cells at the higher concentrations. The observed cell death at high concentrations of NPs could be due to the cell damage caused by the different sized and irregular-shaped NPs with sharp edges during physical interaction. Also, the adsorbed materials and additives in plastics might have enhanced the NPs cytotoxicity [40]. The cells exposed to pristine PSNPs did not show significant cell death as elsewhere reported in different cell lines [40–42]. However, we have recorded a remarkable cell viability increase after 48 h of exposure compared to the control. A similar growth pattern in PSNPs treated cells was observed in the trypan blue assay (discussed below). Likewise, the low concentrations of PENPs, NPs-1, and NPs-2 treatment showed a slight increase in the growth after 48, 72, and 96 h than the control (Additional file 1: Table S2). The observed increase in the cell viability at the low concentration of NPs strengthens the assumption that chemically inert polymers cannot induce cellular toxicity. This scenario is explained below.

### Oxidative stress generation in the HaCaT cells

**Fig. 4:**
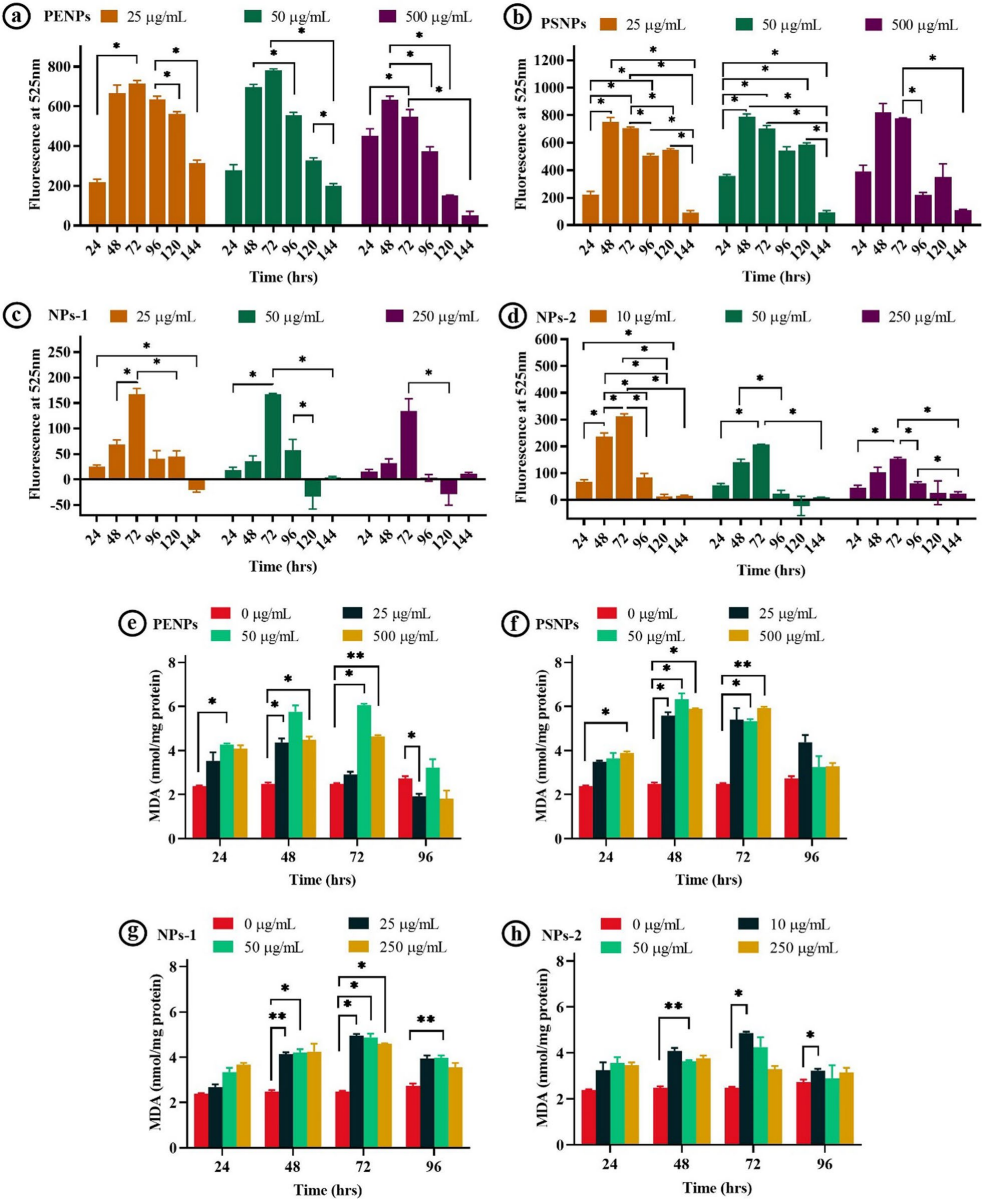
Nano-plastics-induced ROS production and intracellular MDA levels in the HaCaT cells. **a**–**d** ROS activity in HaCaT cells exposed to different concentrations of PENPs, PSNPs, NPs-1, and NPs-2 for 24–144 h, respectively. **e**–**h** MDA levels in the HaCaT cells exposed to different concentrations of NPs at 24, 48, 72, and 96 h, respectively. The values are represented as means ± SEM of two independent experiments. *p < 0.05 significantly different in the ROS production by the NPs concentration versus the exposure time. *p < 0.05, **p < 0.01, ***p < 0.001 significantly different in the MDA levels NPs concentration vs control

In addition to the cell viability assay, we have estimated the unspecific oxidative stress induced by NPs using reactive oxygen species (ROS) assay for six days (Additional file 1: Figure S1). The overall observation showed a difference in the ROS values between the NPs treatments (Fig. 4). The NPs isolated from FS showed significantly low ROS activity compare to the PSNPs and PENPs, which could be due to the increased cytotoxic effect of isolated NPs via physical damage (as mentioned above), denoting high cytotoxicity by direct physical damage over oxidative stress. Though there are similarities among the ROS values of PENPs and PSNPs, only the amorphous PENPs showed cytotoxicity due to cell damage through physical interaction. On the other hand, the PSNPs with spherical shapes and a smooth surface did not cause physical damage on the cell, hence showed a minimal cytotoxic effect. It is also possible that the low level of ROS in cells treated with NPs-1 and NPs-1 could be due to the increased proliferation inhibition as observed in the cell proliferation assay (discussed below). Interestingly, all the NPs showed a maximum ROS level at 48 and 72 h of treatment (Fig. 4a–d), followed by a steady downward trend at 96, 120, and 144 h. The increased ROS level at 48 and 72 h could be due to the high level of NPs interaction and internalization. The observed gradual reduction in the cellular ROS level after 96 h could be due to the defensive action against the ROS or elimination of NPs or even cell destruction [43, 44].

The free radicals produced during oxidative stress could damage the cell membrane’s lipids and fatty acids leading to elevated lipid peroxidation, a primary indicator of cell or organelle damage. Here, the amount of lipid peroxidation in cells was determined from the intracellular malondialdehyde (MDA) level using thiobarbituric acid reactive substances (TBARS) assay. MDA is an end product of lipid peroxidation that reacts with the thiobarbituric acid-trichloroacetic acid (TBA-TCA) complex and produces a detectable coloured substance. The cells exposed to PENPs, PSNPs, NPs-1, and NPs-2 (Fig. 4e–h) showed a concentration and time-dependent increase (*p* < 0.05) in the MDA level compared to the control. However, at 96 h, there was no significant difference (*p* > 0.05) observed in MDA level in the cells treated with high concentrations of PENPs, NPs-1, and NPs-2, and all the PSNPs concentrations (Fig. 4e–h). It is noteworthy to mention that all the tested NPs showed a two-fold increase in MDA production at 48 and 72 h compared to the control.

### Effect of NPs on the antioxidant enzymes

ROS substances are naturally produced in cells during various cellular metabolisms and subsequently eliminated by enzymatic or non-enzymatic antioxidants [45, 46]. When ROS production overwhelms the antioxidant defence system, the equilibrium between prooxidants and antioxidants becomes inadequate, leading to oxidative damage in the nuclei, lipids, and proteins, followed by cell damage [45, 47–49]. Redox homeostasis is the endogenous capacity of cells to deal with continuous challenges generated by electrophiles. An increased accumulation of superoxide anion (O_2_^•−^) and hydrogen peroxide (H_2_O_2_) during oxidative stress could disrupt cellular redox-homeostasis. In order to defend and normalize the ROS stress, cells could produce endogenous antioxidant enzymes. Superoxide dismutase (SOD) is the first line of defence against oxygen‐derived free radicals, and dismutates the O_2_^•−^ into less reactive H_2_O_2_, which is split into H_2_O and O_2_ by catalase (CAT). Herein, the production of SOD in the cells during ROS generation was assayed spectroscopically at 480 nm by the epinephrine auto-oxidation inhibition method. Similarly, the CAT production was assayed from the depletion of hydrogen peroxide.

**Fig. 5:**
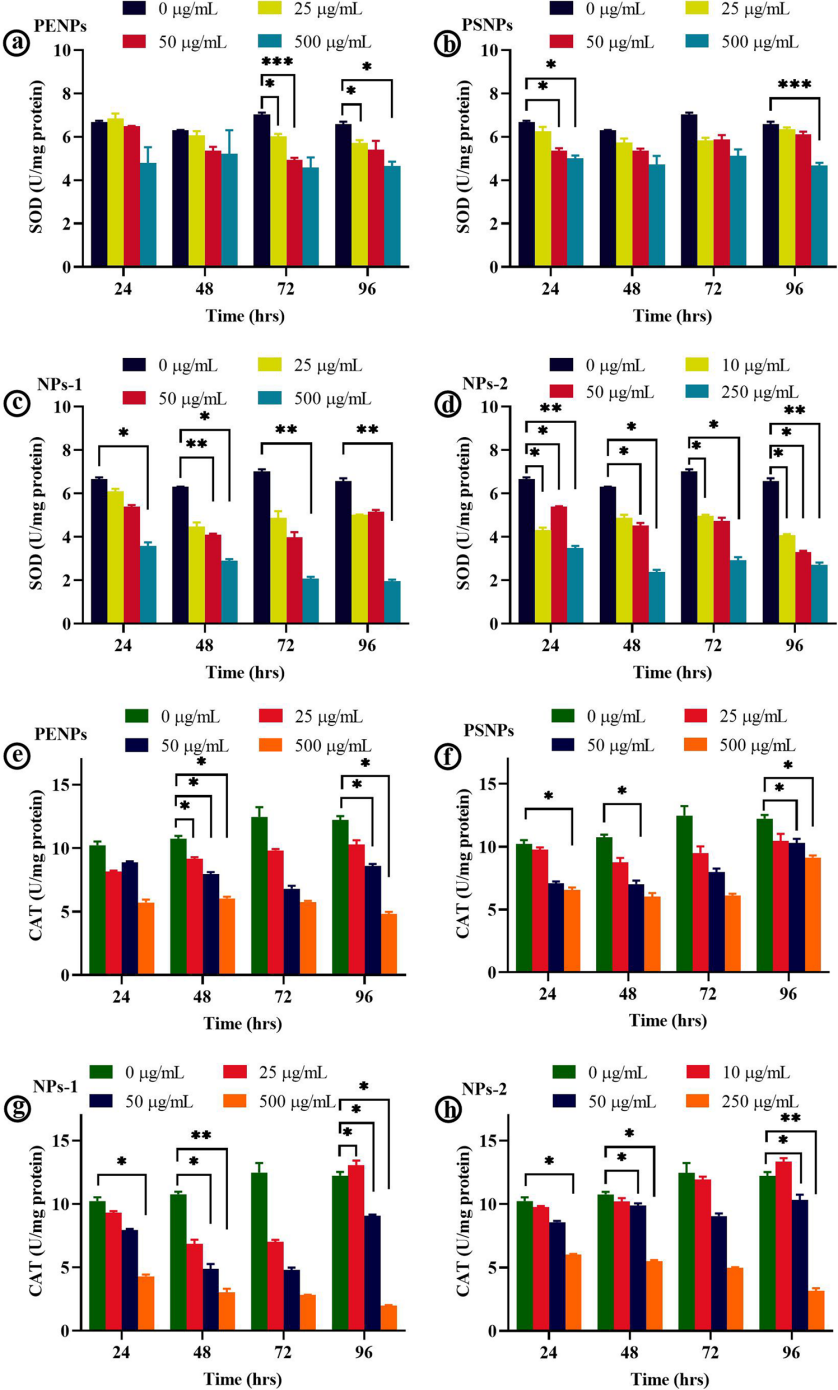
Anti-oxidant enzyme activity in the NPs treated cells. **a**–**d** SOD activity in the HaCaT cells at 24, 48, 72, and 96 h, respectively exposed to different concentrations of NPs. **e**–**h** CAT activity in the HaCaT cells at 24, 48, 72, and 96 h, respectively exposed to different concentrations of NPs. The values are represented as means ± SD of two independent experiments. *p < 0.05, **p < 0.01, ***p < 0.001 significantly different in the enzyme activity of NPs concentration vs control

The chronic long-term treatment of NPs showed a concentration-dependent reduction in the total protein levels compared to control and a time-dependent fluctuation in the SOD and CAT activity. All the NPs (25 and 50 µg/mL) treated cells, except NP-2, displayed a decreasing trend in SOD activity at 24 and 48 h of treatment, followed by a slight increase at 72 and 96 h (Fig. 5a–c). On the other hand, the NPs-2 (Fig. 5d) and high concentration PENPs and PSNPs showed fluctuated SOD levels at different treatment times, while the NPs-1 showed a gradual decrease (Fig. 5c). In CAT activity, all the NPs showed a time and concentration dependent fluctuation in CAT levels (Fig. 5e–h). However, the overall antioxidant enzyme activity remained significantly low in higher concentrations of NPs compared to the control. The observed high level of fluctuation in the SOD and CAT activity of the cells under the long-term treatment with NPs signifies the imbalanced redox-homeostasis in cells. A similar negative correlation between the antioxidants and lipid peroxidation was reported in age-related macular degeneration [50] and heart, lung, liver, and testis of rats [51–54]. It has been postulated that the decrease in the antioxidant activity may be due to the exhaustion of enzymes while detoxifying oxidants [52, 55, 56]. Or else, direct inhibition of antioxidant enzymes by the toxicants [56] and lipid peroxidation [57]. Pigeolet et al. [58] reported that the most important antioxidant enzymes such as SOD, CAT, and GPX (glutathione peroxidase) are susceptible to oxidant metabolites. For instance, SOD is susceptible to H_2_O_2_, and CAT and GPX are susceptible to hydroxyl radicals and superoxide anions [58]. Indeed, these enzymes protect each other from inactivation by the antioxidants. The depletion of one enzyme could increase the oxidants that inactivate the other enzyme, which leads to the exponential production of oxidant molecules and high oxidative stress [58, 59]. It is worth mentioning that reduced antioxidant activity accompanies ROS-mediated cellular responses in the cell.

A low level of ROS acts like signalling molecules that can promote cell proliferation [60], while a moderate level of ROS induces biological responses such as autophagy and senescence, leading to apoptosis and inflammation [61, 62] whereas, the high level of ROS disrupts cells [61–63]. As a result of the above facts, HaCaT cells showed increased viability in PSNPs and at low concentrations of PENPs, NPs-1 and NPs-2 treatment, and increased cytotoxicity at high concentrations of PENPs, NPs-1, and NPs-2. We believe that the PSNPs and low/ sub-lethal concentrations of PENPs, NPs-1, and NPs-2 could stimulate oxidative stress-mediated biological responses in the cells. Before proceeding with this aspect, two important queries need to be answered: (i) how the cells recognize and engulf the NPs, and (ii) why there is a delay in the cellular response against the NPs.

### Mechanism of cell uptake

#### Cellular internalization of NPs

**Fig. 6:**
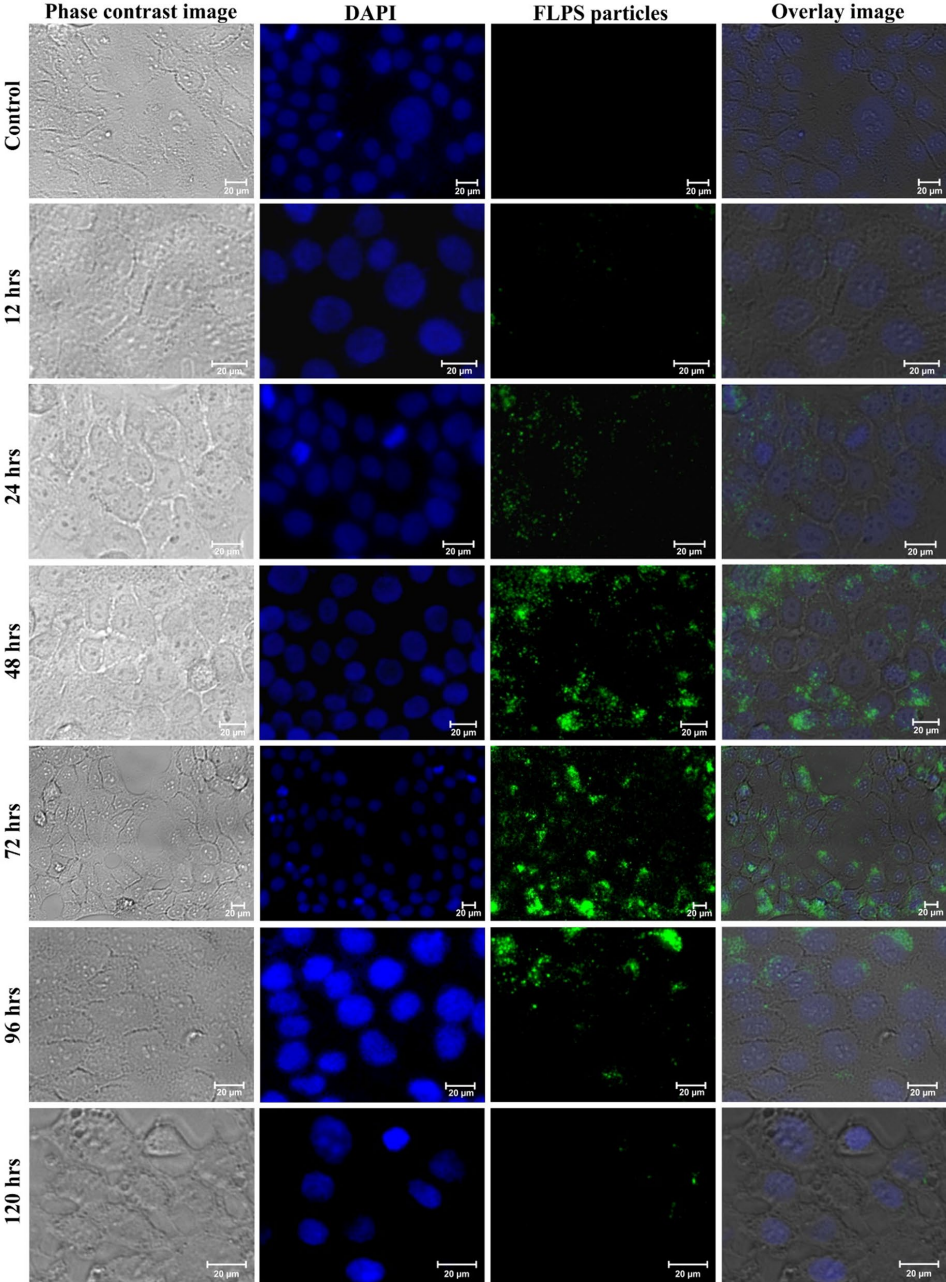
Time-dependent internalization and exclusion of FLPS particles. The phase-contrast, fluorescence, and superimposed images of HaCaT cells exposed to 500 µg/mL concentration of 200 nm sized FLPS particles. For fluorescent imaging, DAPI (*λ*_ex_: 357/44 nm; *λ*_em_: 447/60 nm) and GFP (*λ*_ex_: 470/22 nm; *λ*_em_: 510/42 nm) fluorescent light cubes were used. Image arrangements from top to bottom: control, 24, 48, 72, 96 and 120 h, respectively. Scale bar: 20 μm

To examine the internalization of NPs in the cells, we have treated the keratinocytes with fluorescently labelled PSNPs (FLPS) for 12, 24, 48, 72, 96, 120, and 144 h, and then examined them under a fluorescence microscope. Figure 6 showed a minimum accumulation of FLPS at 12 and 24 h and a maximum internalization at 48 and 72 h of treatment. There was no further increase in the particle accumulation observed at 96, 120 and 144 h, suggesting interruption in the internalization process. Similar ROS-mediated interruption of the endocytosis process was reported in Chinese hamster ovary cells [64] and epidermal cell lines [65]. On the other hand, for the exclusion experiment, the NPs incorporated medium was replaced with a NP free medium after 72 h of exposure. After 96 h, a gradual decrease in the fluorescence intensity in cells (Fig. 6) followed by a complete absence of fluorescence observed at 144 h (Additional file 1: Figure S2). It appears that the gradual reduction of FLPS in cells could be due to the rapid exclusion of internalized FLPS or ROS-mediated cell death or both. As a result of the increased uptake of NPs at 48 and 72 h treatment (Fig. 6), the keratinocytes presented an elevated level of ROS and reduced antioxidant activity. The observed reduction in the ROS level after 72 h of treatment could be either due to the rise of defensive antioxidant activity in cells (Fig. 5), NPs removal from the cells, blocking of NPs internalization, or cell death. The result observed in the FLPS experiment signifies that the NPs internalization does not occur immediately but requires some time. However, further investigations are needed for a better understanding of the mechanism of uptake.

#### Protein-corona formation on the NPs

We assume that the keratinocytes might have recognized the NPs as a foreign substance and prevented its entry for about 12 h, but later, due to the surface modification of NPs, the HaCaT cells might have recognized and internalized it. As described in our previous study, the biological macromolecules, especially proteins, tend to adsorb on NPs surfaces called protein corona, which in turn mimic protein aggregates [15]. To validate the corona formation on NPs under DMEM, the NPs were introduced into the medium and incubated for 6, 12, and 24 h and then examined under HR-TEM [15]. The TEM micrographs (Additional file 1: Figure S3) showed about 10–200 nm-sized corona formation by proteins and smaller biomolecular aggregates on PENPs (Additional file 1: Figure S3a) and PSNPs (Additional file 1: Figure S3b). On the other hand, the NPs-1 (Additional file 1: Figure S3c) and NPs-2 (Additional file 1: Figure S3d) showed corona-mediated aggregation in correlation with the DLS results, where we observed a three to fourfold increase in the particle size. After 24 h, the total number of aggregated particles was high compared to 12 and 6 h of exposure, but the average size remained < 700 nm.

#### Protein-corona-mediated internalization of NPs

**Fig. 7:**
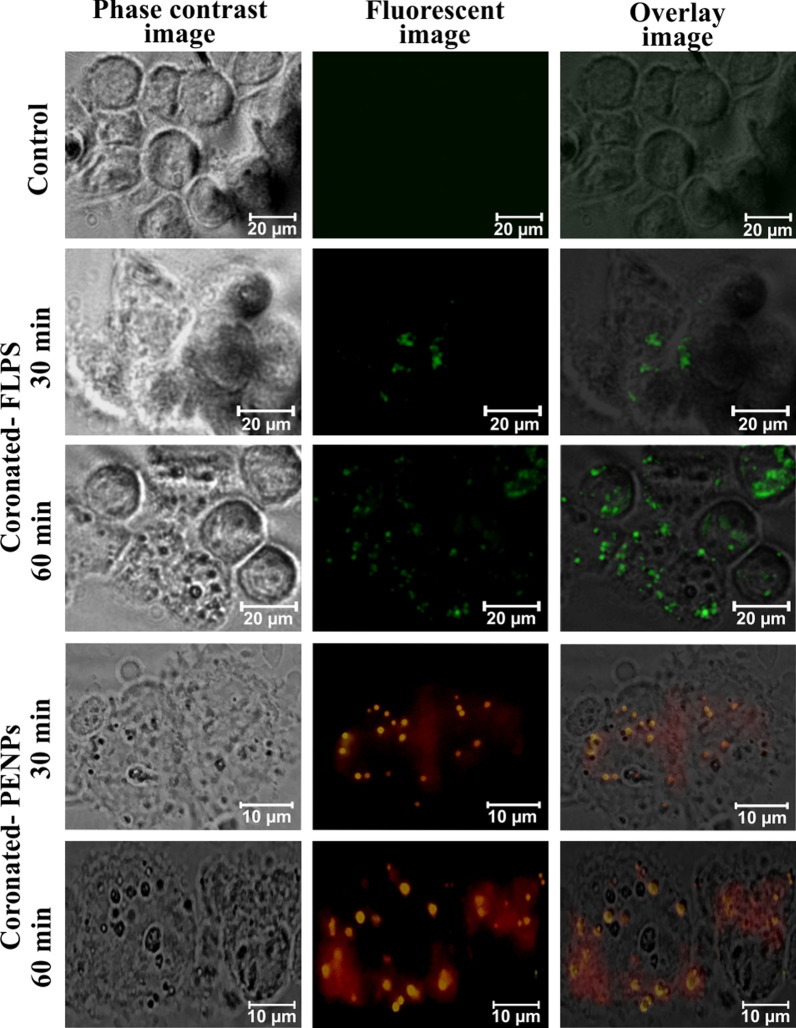
Keratin-corona mediated rapid internalization of NPs in HaCaT cells. The phase-contrast, fluorescence, and superimposed images of HaCaT cells exposed to FLPS and Nile red stained PENPs particles. For the fluorescent imaging of coronated-FLPS and -PENPs, FITC fluorescent filter and I3 filter cube was used, respectively. Image arrangements from top to bottom: control, FLPS exposed for 30 min and 60 min, PENPs exposed for 30 and 60 min, respectively. The fluorescent-NPs without keratin-corona was used as a control

Generally, plastic particles tend to adsorb organic and inorganic substances quite rapidly, and the rate of corona formation and corona thickness is directly proportional to the availability of biomolecules. For example, in human serum albumin solution and human blood plasma, a multi-layered protein-corona of a few hundred-nanometre radii was formed on PSNPs within 2 h [15]. We strongly suspect that the observed delay in the protein-corona formation in DMEM could be due to the solution properties, competitive exchange of media components, or a shortage of macromolecules. As a result of the delayed corona formation, we observed a limited FLPS uptake by keratinocytes for up to 12 h (Fig. 6 and Additional file 1: Figure S2). As mentioned above, under the in-vivo system, corona formation and cellular uptake could occur within a few hours of exposure. To demonstrate the significant role of protein-corona in NPs recognition and internalization, we have prepared fluorescent PENPs using Nile red stain (Additional file 1) and added it into the keratin solution (0.2%) for 1 h to produce keratin-corona (Additional file 1: Figure S4). Likewise, the keratin-coronated green fluorescent FLPS was prepared, purified, dispersed in DMEM, and exposed to the HaCaT cells (Additional file 1). After 30 min and 60 min of incubation, the cells were harvested and examined under a fluorescent microscope. Figure 7 showed a rapid internalization of fluorescent-PENPs and -PSNPs compared to the cells treated with corona-free-NPs (Fig. 6), where the uptake occurred only after 12 h. This observation certainly proved that the adsorption of proteins and other molecules on the NPs provided a cloaked identity, and subsequently triggered the cell recognition and internalization of NPs. To maintain uniformity in the corona formation on NPs and corona-mediated effect on cells, we preferred the protein-corona formation under DMEM throughout this study.

#### Macropinocytosis and lysosomal action

**Fig. 8:**
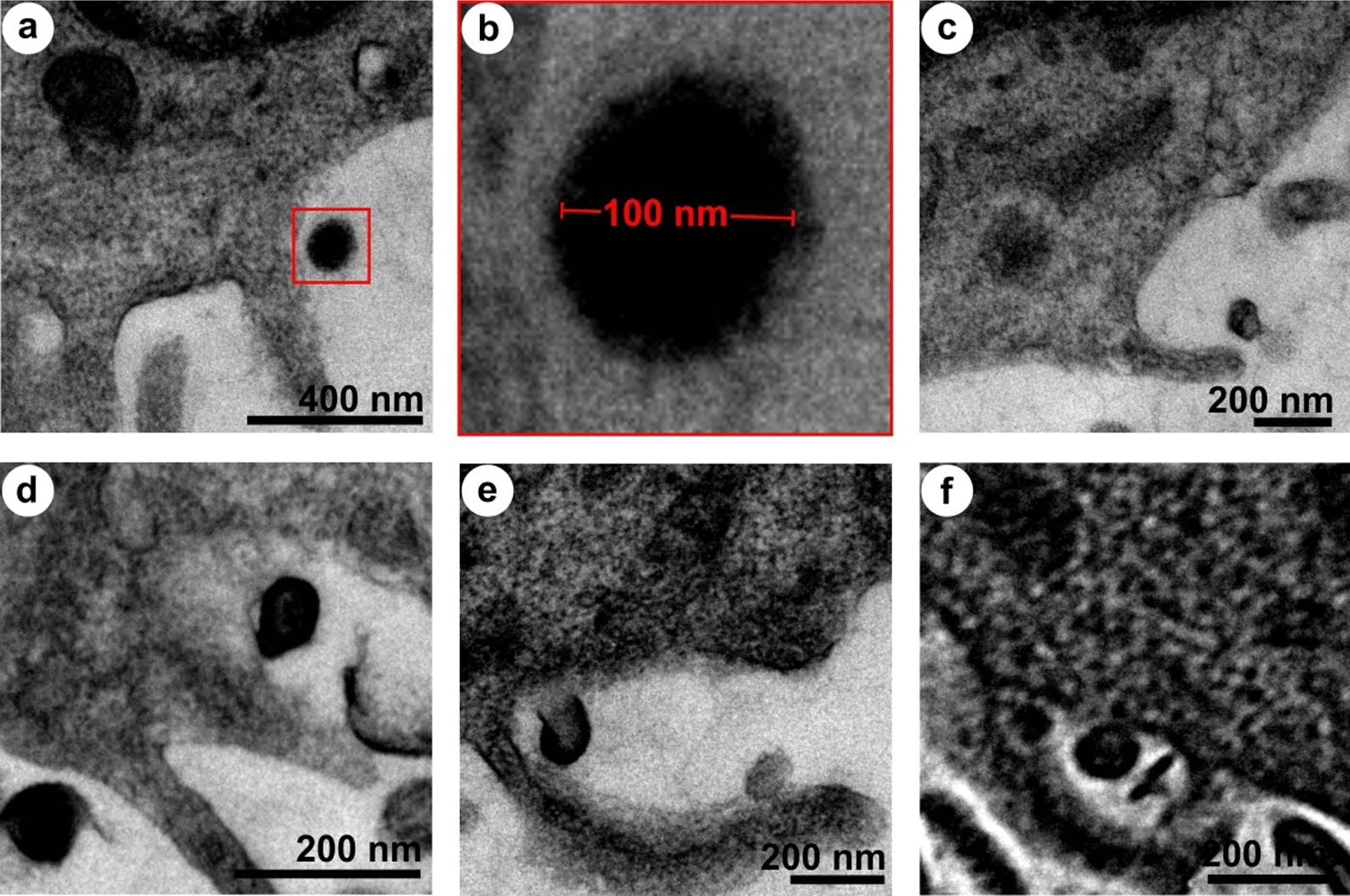
Mechanism of NPs internalization in HaCaT cells. **a** Binding of coronated NPs on keratinocytes surface, **b** magnified view of NPs on the cells surface, **c**, **d** pinocytic cups/large membrane ruffles formation, **e** membrane ruffles folding back on the cell surface along with the coronated NPs and **f** membrane surrounded intracellular compartment

**Fig. 9:**
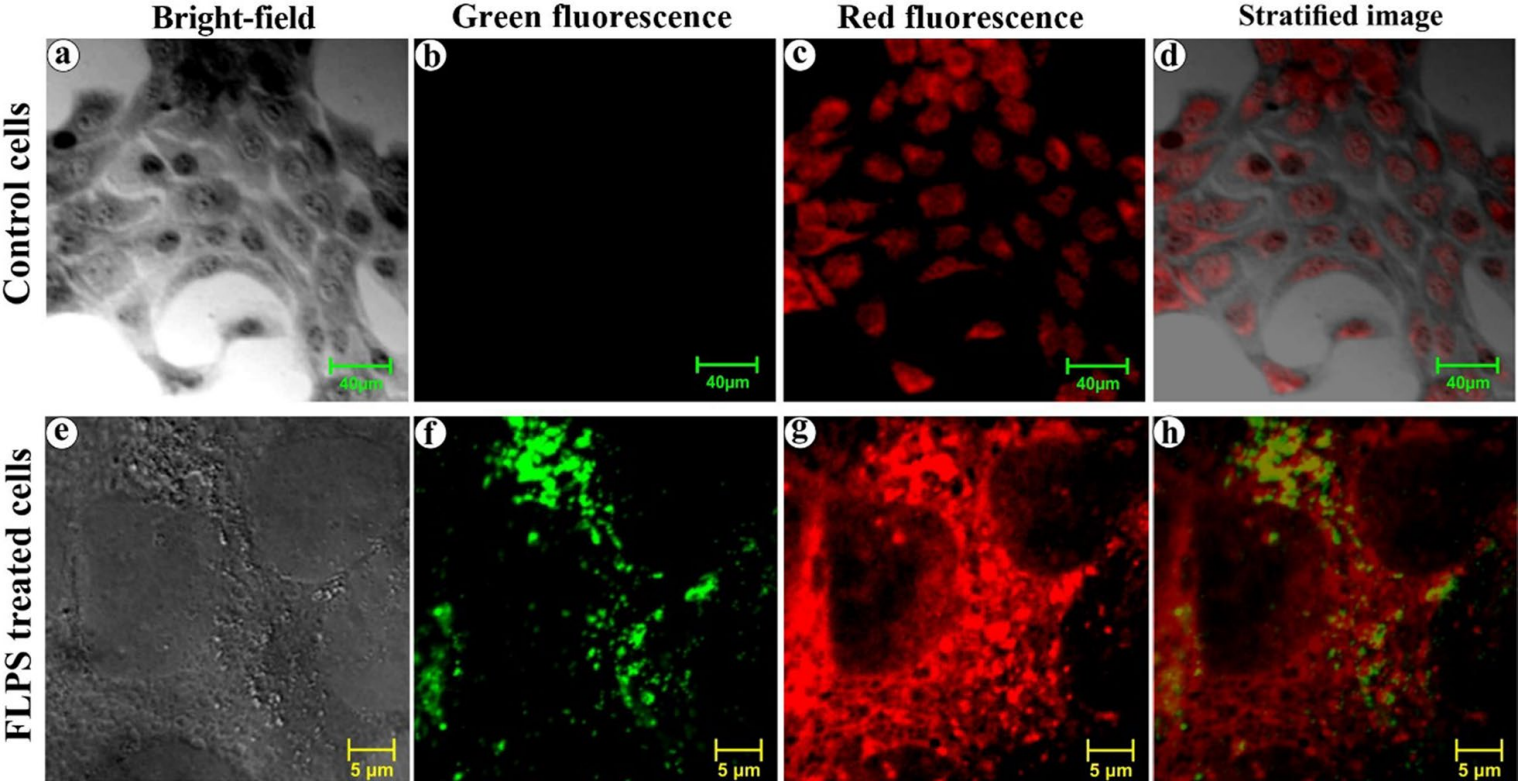
Lysosomal activity on the internalized NPs. Confocal laser scanning images of control (**a**–**c**) and FLPS treated (**e–g**) HaCaT cell, respectively from bright-field, green (*λ*_ex_: 458 nm; *λ*_em_: 485 nm) and red (*λ*_ex_: 540 nm; *λ*_em_: 610 nm) fluorescent channel. **c**, **g** neutral red (a lysosomal probe) stained lysosomes. **f** green fluorescent-FLPS located within the macropinosomes. **d**, **h** superimposed images of the bright-field, green, and red fluorescent images. Scale bar- 40 µm (control) and 5 µm (treated cells)

As a result of protein-corona formation on the NPs, the cells might have recognized the coronated-NPs as protein aggregates and internalized them via macropinocytosis, an ideal mechanism towards protein aggregates [66–69]. Yet, the macropinocyte activation mechanism by protein aggregates remains unidentified [70]. To observe the internalization process, the cells were treated with PSNPs for 48 h, fixed, harvested, embedded, and sectioned before the HR-TEM analysis (Fig. 8). Herein, the spherical NPs were applied to avoid ambiguity in the visual sorting of NPs and cell structures under electron microscopy. The electron micrographs showed an attachment of coronated-NPs on the cell surface (Fig. 8a, b) and the formation of pinocytic cups, or large membrane ruffles (Fig. 8c, d). Additionally, the folding back of membrane ruffle onto the cell surface with the coronated NPs (Fig. 8e) and the formation of a membrane surrounded intracellular compartment were seen (Fig. 8f) [71]. After understanding the process of NPs uptake, we have studied the lysosome fusion with macropinocytes and macropinolysosomal activity using a confocal laser scanning microscope (CLSM). For this experiment, we used the FLPS emitting green fluorescence (*λ*_ex_ 458 nm and *λ*_em_ 485 nm), and neutral red (a lysosomal probe) [72] that emits red fluorescence (*λ*_ex_ 541 nm and *λ*_em_ 610 nm) [73]. Under CLSM, the cells treated with NPs for 48 h showed intense green and red fluorescence from the FLPS within the macropinosomes (Fig. 9f) and neutral red stained lysosomes, respectively (Fig. 9g). The stratified images (Fig. 9h) of red and green fluorescence depicted the formation of macropinolysosomes (yellow colour). Additionally, the accumulated lysosomes (red fluorescence) around macropinosomes indicate a possible fusion attempt. The formation of macropinolysosomes represents the commencement of degradation of protein-covered NPs. Red fluorescence signals measured from the neutral red stained lysosomes in the NPs treated cells (Additional file 1: Figure S5b) showed 3.5-fold higher mean fluorescence intensity (MFI) when compared to the untreated cells (Additional file 1: Figure S5a), denoting increased production of lysosomes in the cell due to the NPs internalization. The lysosomal activity on the internalized NPs was found to be ~ 20% (Additional file 1: Figure S5c) and about 80% of the macropinosomes are fused with the lysosomes (Additional file 1: Figure S5d) (refer Additional file 1 for more details).

#### The fate of NPs in macropinolysosomal activity

Generally, cells eliminate the undigested or toxic substances through exosomes. But during adverse conditions or cell death, these substances are released nakedly. To examine the morphological alterations in the NPs by lysosomal action, we have treated the cells with PSNPs for 48 h, washed twice with PBS (phosphate-buffered saline), and then incubated with a fresh NPs-free medium. After 24 and 48 h of incubation, the NPs released in the culture medium were separated and examined under HR-TEM (Additional file 1: Figure S6). The electron micrographs showed partly damaged (Additional file 1: Figure S6c-f), disintegrated (Additional file 1: Figure S6g-l), and enlarged NPs (Additional file 1: Figure S6m-o) due to various enzymatic actions in the macropinolysosomes. The observed corrosion in PSNPs (Additional file 1: Figure S6g-l) under HR-TEM suggests that the NPs might have disintegrated during the digestion process. It could pave the way for the leaching and release of styrene molecules and additives into the cells. The release of styrene molecules from PSNPs treated cells was detected using gas chromatography along with the styrene standard (Additional file 1: Figure S7) [73], yet it has to be studied further to quantify the molecular release with other NPs as well. The observed corrosion of PSNPs and release of styrene molecules within the cells raises concerns about the emission of endocrine-disrupting additives, such as bisphenol A, nonylphenol, and octylphenol present in plastic products [74–77]. All the above results revealed that the surface modification of NPs (protein-corona formation) eventually triggers the engulfment process followed by lysosomal action, particle degradation, and oxidative stress.

### The cytoprotective activity in keratinocytes post-NPs internalization

#### Inhibition of cell proliferation

**Fig. 10:**
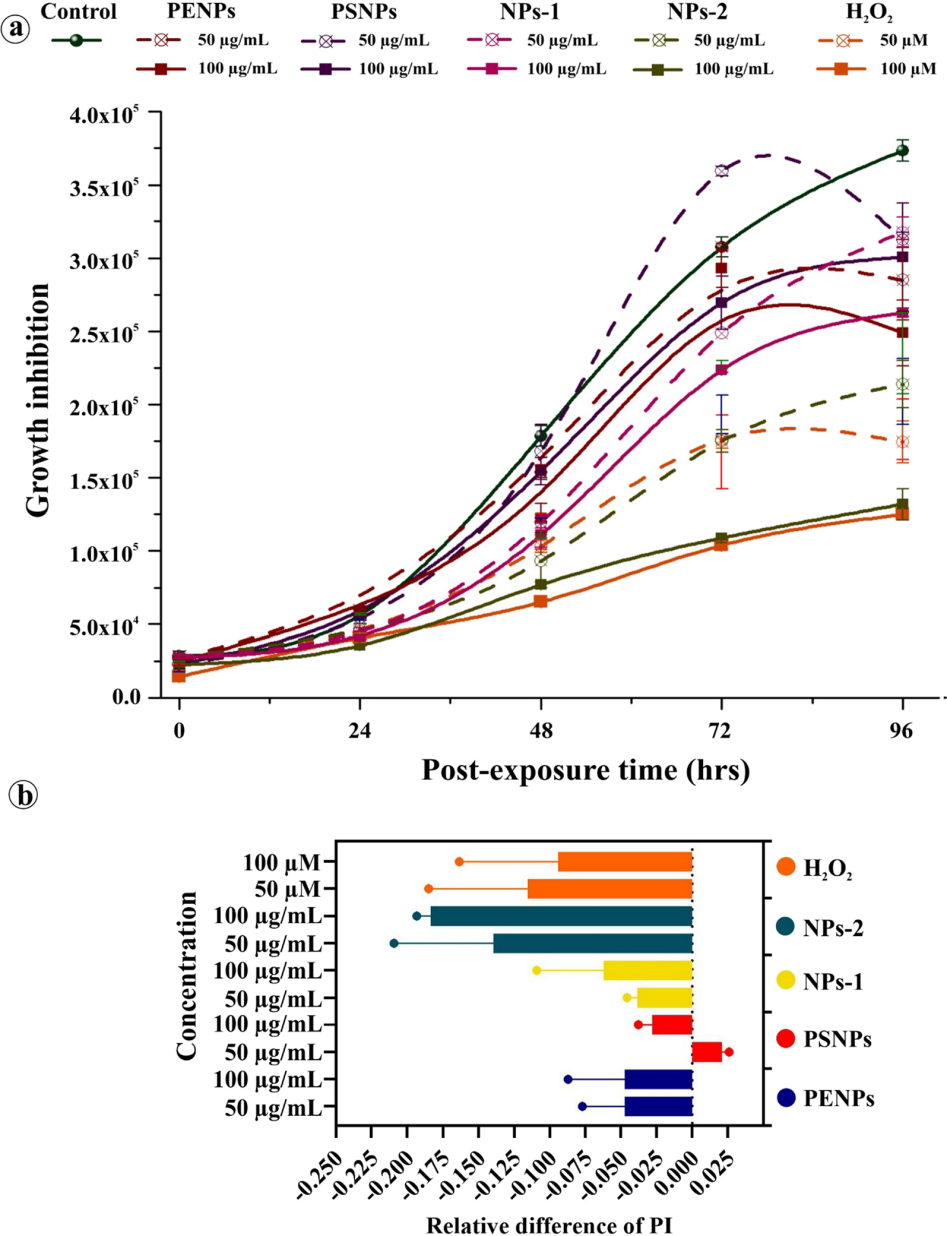
The effect of a single-dose exposure to lethal and sub-lethal concentrations of NPs on the proliferation of HaCaT cells. **a** The NPs induced growth inhibition in the HaCat cells exposed to PENPs (50 and 100 µg mL^−1^), PSNPs (50 and 100 µg mL^−1^), NPs-1 (50 and 100 µg mL^−1^), NPs-2 (50 and 100 µg mL^−1^) and H_2_O_2_ (50 & 100 µM). **b** The relative difference in the proliferation index among the NPs. Error bars indicate standard error

The absence of significant cytotoxic effect in cells at the low concentration of NPs and all PSNPs concentrations indicates the possible activation of cytoprotective mechanisms, especially inhibition of cell proliferation, senescence, and autophagy. To demonstrate the cytoprotective events in keratinocytes, we have treated the cells with lethal- and sub-lethal-doses of NPs for 48 h. After achieving the optimum internalization of NPs, the medium containing NPs was removed and replaced with a fresh, NPs-free medium. Then, the cell proliferation inhibition in the NPs internalized cells were determined from the total number of viable cells at every 24 h interval for four consecutive days compared to the control. The NPs internalized cells showed a concentration-dependent decrease in the cell viability (Fig. 10a). However, under microscopic examination, minimal accumulation of trypan blue in the cells was observed, denoting that the cells are at the early stage of proliferation inhibition but remain metabolically active and viable. Among the tested NPs, NPs-2 showed significant growth inhibition, followed by NPs-1, PENPs, and PSNPs. The cell proliferation index calculated from the relative difference between the pre- and post-internalization of NPs exhibited NPs concentrations- and physicochemical properties-dependant inhibition in the cell proliferation (Fig. 10b). The results are correlated with the cytotoxic assay and signified a single dose of NPs could cause a cytostatic effect in cells.

#### Cellular senescence and autophagy

**Fig. 11:**
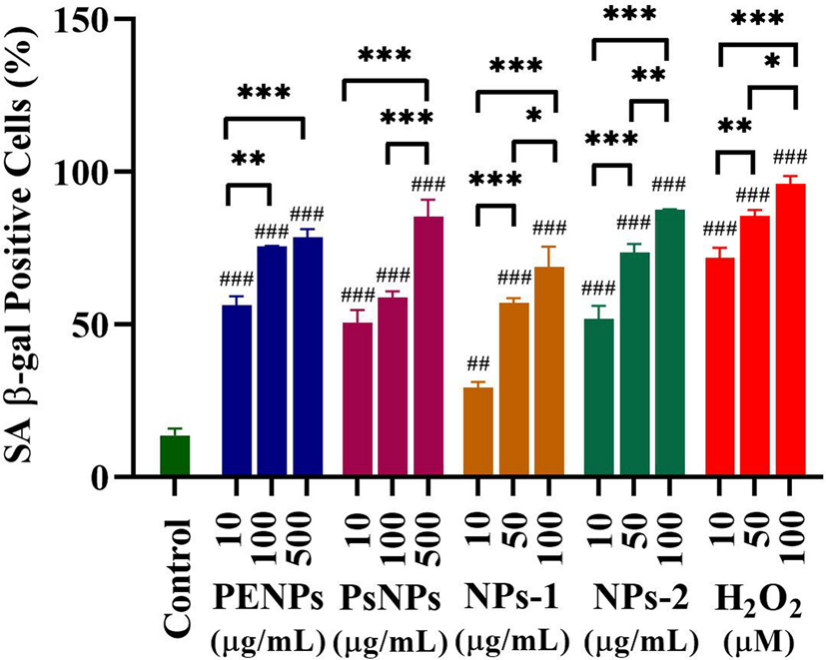
Quantification of SA-β-gal positive cells that are treated with PENPs (10, 100 & 500 µg mL^−1^), PSNPs (10, 100 & 500 µg mL^−1^), NPs-1 (10, 50 & 100 µg mL^−1^), and NPs-2 (10, 50 & 100 µg mL^−1^) and H_2_O_2_ (10, 50 & 100 µM) for 48 h and then incubated with fresh, NPs-free medium. Micrographs were obtained with phase-contrast microscopy. Totally 250 cells scored from five images were expressed as the percentage (mean ± SEM) of positive cells. * *p* < 0.05, ** *p* < 0.01, *** *p* < 0.001 significantly different from the same NPs concentration. # *p* < 0.05, ## *p* < 0.01, ### *p* < 0.001 significantly different from the control

The senescence is the permanent halt in cell growth, which could resist apoptotic death for a long period [78, 79]. The abnormal accumulation of β-galactosidase has widely been reported in senescent cells, which allows the senescence-associated β-galactosidase (SA-β-gal) to be an important biomarker for cellular senescence. However, the underlying mechanism in the origin of SA-β-gal activity and its role in senescence and aging are still unknown. The HaCaT cells treated with PENPs (10, 100 & 500 µg/mL), PSNPs (10, 100 & 500 µg/mL), NPs-1 (10, 50 & 100 µg/mL), NPs-2 (10, 50 & 100 µg/mL) and H_2_O_2_ (10, 50 & 100 µM) for 48 h (for maximum NPs uptake) were washed and incubated in NPs-free medium for 24 h and then stained for SA-β-gal activity (Additional file 1: Figure S8). The microscopic observations showed a concentration-dependent increase in the SA-β-gal-activity in the PENPs, PSNPs, NPs-1, NPs-2, and H_2_O_2_ treated cells compared to the untreated cells (Fig. 11 and Additional file 1: Table S3). The mean percentage of SA-β-gal positive cells showed a significant difference (*p* < 0.05) within the NPs concentrations and with control. Additionally, the NPs and H_2_O_2_ treated cells showed typical senescent morphologies such as enlarged, flattened cells and increased accumulation of cytoplasmic granules [80, 81].

**Fig. 12:**
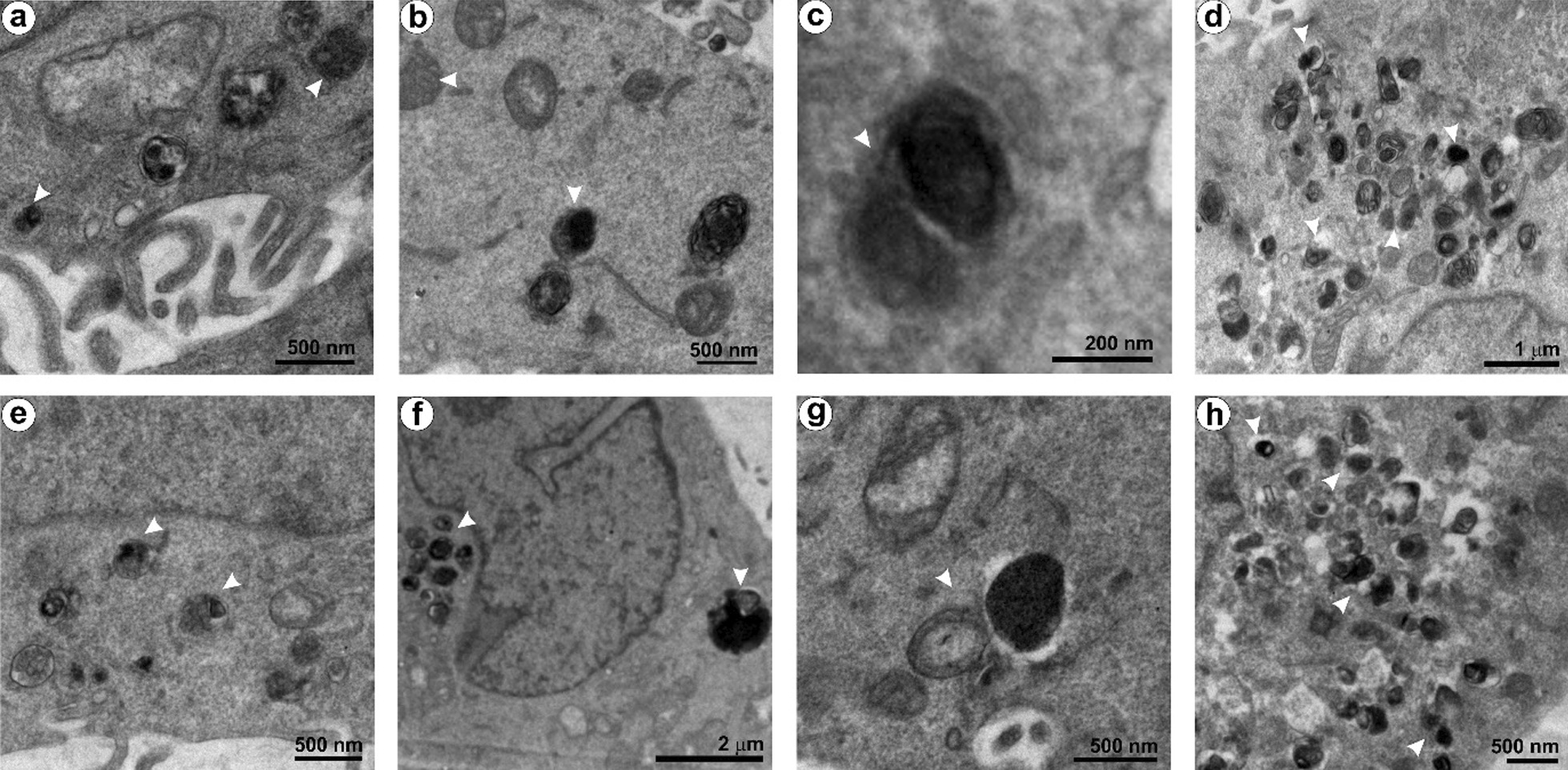
Accumulation of autophagy structures in the keratinocytes treated with a single-dose of NPs for 48 h and then incubated with fresh, NPs-free medium. **a–d** Ultrastructure of HaCaT cells exposed to NPs-1. **e**–**h** Ultrastructure of HaCaT cells exposed to NPs-2. White arrow heads indicate the autophagic structures

Besides, the cellular senescence may lead to chromatin modification, metabolic refinement and high autophagy activity, and pro-inflammatory-secretome production in cells [78]. Among the phenotypes, autophagy, a genetically regulated bulk degradation process, was detected in the NPs treated cells (Fig. 12). Autophagy is a cell survival mechanism that degrades the damaged cytoplasmic organelles and long-lived proteins using lysosomes. [82, 83]. Autophagy could facilitate senescence or limit cell damage, or delay apoptosis, which allows the cell to recover normal function [84–86]. The keratinocytes treated with NPs-1 (Fig. 12a–d) and NPs-2 (Fig. 12e–h) showed a series of autophagy structures under HR-TEM. These structures revealed an active engulfment of damaged organelles by phagophore that becomes an autophagosome. After maturation, the autophagosome fuses with the lysosome (termed autophagolysosome/ autolysosomes) for active digestion. The observed proliferation inhibition, senescence, and autophagy activity in the NPs treated cells envisages the homeostasis against a low level of oxidative stress. The results further emphasized that all the ROS-mediated molecular pathways may be interconnected [84, 87]. However, a high level of cytoprotective events can trigger inflammation and apoptosis [61, 62, 85, 87].

Recent studies on the cytotoxicity of MPs and NPs in human and animal models showed that low concentrations of plastic particles produce oxidative stress in cells, and high concentration plastic particles cause cytotoxic [88, 89]. Additionally, in this study, we have demonstrated the NPs concentration-dependent regulatory activity and cytoprotective and cytotoxic effects in HaCaT cells. This study presents three lines of evidence that are essential to close the existing knowledge gap in the cell uptake and response against NPs; firstly, plastic particles adsorb protein molecules and mimic protein aggregates, thereby triggering and accelerating the internalization process. Secondly, the internalized NPs undergo lysosomal activity, which damages the NPs and leads to the release of plastic molecules and additives within the cells, subsequently accelerates oxidative stress. Finally, the ROS stress down-regulates cell proliferation and inhibits cell growth leading to premature aging, autophagy, or ROS-induced cytotoxic effect. To conclude, the continuous use of NPs and MPs particles incorporated cosmetics over a prolonged time may result in premature aging of skin cells.

## Conclusion

Despite the wide use of cosmetic products containing a high concentration of NPs/MPs, not many studies have demonstrated the NPs internalization, accumulation, and toxicity in skin cells. Hence, in the present study, we have used HaCaT cells to evaluate the potential risk of single- and long-term-doses of NPs on human skin. To the best of our knowledge, this is the first study that demonstrates the protein-corona-mediated entry of plastic particles, lysosomal-mediated disintegration of NPs, and additives release in cells. We have also presented the NPs concentration-dependent ROS production, cytoprotective and cytotoxic events in cells. The observed proliferation inhibition, senescence, and autophagy activity in cells evidence the homeostasis attempt via cytoplasmic and organelle turnover against a low level of oxidative stress. However, at the high level of cytoprotective events, especially autophagy, may cause autophagic cell death.

Although the concentration of NPs exposed to humans in every single use has not been precisely calculated, the concentrations used herein are still an order the magnitude greater than realistic human exposure. We believe that the results obtained from the 2D monolayer culture model are preliminary, but novel evidence on keratinocyte's physiological responses against NPs. Even though 2D cultures have many limitations and are highly variable from the natural architecture, most of the physiological events and molecular pathways taking place within the cells were elucidated using a 2D culture technique. For more realistic exposure and dose analysis at the physiological and molecular level, and to validate additional risks of applying polymer NPs incorporated cosmetics on skin cells and NPs disintegration within the cells, a 3D skin model is needed [90, 91]. Further studies are required to analyze the influence of cosmetic ingredients on the protein-corona formation, define the protein-corona-mediated recognition and macropinocytosis process, and determine the efflux action. Collectively, these findings will shed light on the effects of plastic particles on human cells and might seed further studies. At the outset, our results emphasize an immediate action to curtail the use of NPs/MPs and demand the use of more natural and sustainable ingredients in CPCP.

## Materials and methods

### Materials, reagents, and cell culture

Polyethylene pellets (Polysciences, Inc., Warrington, PA), polystyrene NPs (PSNPs- 100 nm) (Catalog # 108821–10, Corpuscular Inc., NY, USA), and fluorescently labelled polystyrene NPs (FLPS- 200 nm) (Fluoresbrite® Yellow Green 0.20 µm Microspheres, Polysciences, Inc.) were procured commercially and used in this study. The 2', 7'-Dichlorofluorescin diacetate (DCFDA) was purchased from Sigma Aldrich (St. Louis, MO). The DAPI (4', 6-Diamidino-2-Phenylindole, Dihydrochloride) and MTT (3‐ [4, 5‐dimethylthiazol‐2‐yl] ‐2, 5‐diphenyl tetrazolium bromide) were procured from HiMedia, India. The Senescence β-Galactosidase Staining Kit (#9860, CST) was acquired from Cell Signalling Technology (Beverly, MA, USA). Collagen and keratin were extracted from fish scales [92] and human hair fibres [93], respectively, and used in this study (Additional file 1).

The nano-sized PENPs were prepared from the subsequent breakdown of polyethylene pellets using a stainless-steel grain grinder mill, homogenizer, and ultra-sonicator. Briefly, the powder collected from the grinder mill was sieved using a stainless steel sieve (75 µm), and the sieved powder (10 mg) was homogenized in deionized water (50 mL) until a pale smoke colour solution formed. The solution was then separated and sonicated for 2 h. The pale solution was filtered subsequently using 20–25 μm, 2.5 μm, and 0.4 μm filters and then quantified for further studies. Before each experiment, the PENPs dispersion solution was sonicated for 30 min. The stability, zeta-potential, Raman shift, and morphometry of PENPs were analysed as described below.

The human keratinocytes (HaCaT cells) were cultured in DMEM (HiMedia) containing glucose (4.5 g/l), sodium pyruvate, FBS (10%), L-glutamine (2 mM), penicillin (100U/mL), streptomycin (100 µg/mL) and amphotericin B (0.25 µg/mL) and maintained under a 95% humidified incubator at 37 °C with 5% CO_2_. The same cultural conditions were maintained throughout the study.

### Isolation of NPs from face scrubs

About 0.2 g samples from 10 replicates of each FS were diluted separately in 10 mL of filter-sterilized, surfactant-free ultra-pure hot water (Additional file 1: Figure S9) and subsequently filtered through 20–25 μm (Grade 2 Whatman®), 2.5 μm (Grade 41 Whatman®), 0.4 μm (Whatman® 110607, Nuclepore membrane filter) and 0.2 μm (Whatman® 7182-014, Cellulose nitrate membrane filter). Final filtrates obtained from a 0.2 μm filter were pooled together with the respective FS and quantified. The spectral and morphological features of the dried NPs were analysed using Fourier Transform Raman Spectroscopy (FT-Raman) (PerkinElmer 1600 instrument, USA) and HR-SEM (Carl Zeiss Evo 18 SEM, Germany). The dispersion, stability, and size distribution of the NPs in culture medium (DMEM) were measured using the Malvern Dynamic Light Scattering instrument (Malvern, UK) in triplicates with 20 runs each.

### Adsorption of NP particles on the keratin layer

The microscopic glass slides were cut into 1cm^2^ glass pieces, immersed in detergent solution, sonicated, rinsed with distilled water, and cleaned with freshly prepared 3:1 HCl: HNO_3_ (aqua regia) for 30 min and then thoroughly rinsed using ultra-pure water. Each glass piece was immersed in a 0.1% collagen solution overnight under a shaker incubator. After incubation, the glass pieces were carefully removed, wiped at one side, placed into a Petri dish to drain the excess moisture. About 100 µL of keratin solution (0.5%) was drop coated on the collagen layer and dried at room temperature (Additional file 1: Figure S10). Herein, the keratin concentration was determined based on the estimate of Chao and Nylander–French [94] that the average total keratin mass of stratum corneum in males and females was more than 400 µg/cm^2^. The keratin-coated glass pieces were immersed in the PENPs suspension (100 µg/mL) for 2–3 min under wrist-action shaking at room temperature. After incubation, the glass pieces were carefully removed and subsequently washed by 8–10 dipping in ultra-pure water for 30 s. Another set of glass pieces were washed twice (8–10 dipping/ wash) with a 30-s interval to check the sturdy adsorption of NPs on the protein layer. The washed (1 or 2 times) and unwashed glass pieces were dried, gold-coated using sputter coater, and then observed under field-emission scanning electron microscope (FE-SEM). The same procedure was carried out for PSNPs, NPs-1, and NPs-2.

### NP particles interaction with keratinocytes

#### Chronic long-term cytotoxicity assay

HaCaT cells (1 × 10^4^) were seeded in 96 well plates and incubated. After achieving confluency, the DMEM was replaced with fresh medium containing different concentrations of PENPs (25, 50, 100, 250, 500 µg/mL), PSNPs (25, 50, 100, 250, 500 µg/mL), NPs-1 (25, 50, 100, 250, 500 µg/mL) and NPs-2 (5, 10, 50, 100, 250 µg/mL) into respective wells and incubated. The working concentrations of all the NPs in the culture medium were prepared without altering the concentration of the medium components from the stock solutions of PENPs (1 mg/mL), PSNPs (25 mg/mL), NPs-2 (2 mg/mL), and NPs-1 (2 mg/mL), vortexed vigorously for 10 min and used immediately, or eventually, depending on the experiment. Every 24 h, the cell viability was measured using the MTT assay. Herein, the experiment was conducted for six consecutive days because most in vitro studies on MPs or NPs were conducted for less than 48 h, but in real-world events, there might be chronic long-term exposure. Additionally, the NPs incorporated DMEM medium was maintained under culture conditions and used as a replacement medium (every 24 h) for all the experiments unless otherwise stated.

#### Determination of intracellular oxidative stress

HaCaT cells (1 × 10^4^) were seeded in 96-well plates and incubated. The ROS production in HaCaT cells exposed to the above-used concentrations of PENPs, PSNPs, NPs-1, and NPs-2 was measured for six consecutive days using the DCFDA assay. Briefly, the NPs treated cells were washed twice with PBS (phosphate-buffered saline) at every 24 h intervals and about 10 µM DCFDA was added and incubated for 60 min. In the event of ROS production, the non-fluorescent DCFDA oxidized into green fluorescent 2′, 7′–dichlorofluorescein (DCF). The level of fluorescence is directly proportional to the oxidative stress in the cells [95]. After 60 min, the cells were washed with PBS twice and measured for fluorescence using a spectrofluorometer (JASCO FP-8300, Japan) with excitation and emission wavelengths of 495 and 525 nm, respectively. Additionally, hydrogen peroxide (25, 50, 100, 250, 500 µM) was used as the positive ROS indicator.

#### Determination of oxidative stress marker

HaCaT cells (1 × 10^5^) were seeded in a culture dish and incubated to achieve confluence, then the cells were treated with different concentration of PENPs (25, 50, 500 µg /mL), PSNPs (25, 50, 500 µg /mL), NPs-1 (25, 50, 500 µg /mL), NPs-2 (10, 50, 250 µg /mL). At every 24 h interval for four consecutive days, the spent medium with NPs was withdrawn and discarded. The cells were washed three times with cold PBS, detached by scraping, suspended subsequently in sodium phosphate buffer (10 mM, pH 7.5), and then homogenized. To the homogenate, 1% Triton X-100 was added and incubated on ice for 10 min and then centrifuged (5000 g) at 4° C for 10 min. The protein content was estimated from the clear supernatant by the Bradford method. Lipid peroxidation was estimated through the thiobarbituric acid reactive substance concentration (TBARS) [96]. Briefly, the protein molecules were precipitated from the cell homogenate using TCA (10% w/v). The clear supernatant obtained from centrifugation was mixed with 0.67% TBA (w/v), heated for 30 min at 95 °C in a water bath. Herein, the reaction between TBA and oxidative degradation of lipid products produced a red complex with an absorbance maximum at 535 nm. Here, 1, 1, 3, 3-tetra methoxy propane was used as the standard, and the lipid peroxidation was presented as nmol/mg protein.

#### Determination of antioxidant enzyme activity

The amount of epinephrine auto-oxidation inhibition by SOD was used to determine the total SOD activity [97]. Briefly, 50 µg of protein was added to 500 mM sodium phosphate buffer (pH 10.2) and epinephrine (1 mM). At pH 10, epinephrine rapidly undergoes auto-oxidation that produces pink-colour adrenochrome, which was measured at 480 nm using a microplate reader (Biotek, USA.) The amount of enzyme required to cause 50% inhibition was defined as 1 unit of enzyme activity, and the total SOD activity was expressed as units/mg protein. Similarly, for catalase activity, 50 µl of protein extract was added to sodium phosphate buffer (50 mM, pH 7.0) and hydrogen peroxide (100 mM) mixture, shaken, and then incubated under dark for 10 min at 37 °C. The changes in the absorbance at 240 nm were measured against the blank, and the CAT activity was expressed as units/mg protein [98].

### Degree of NPs cellular internalization and exclusion

#### Determination of NPs internalization

To study the NPs internalization, retention, and elimination, we used green fluorescent FLPS particles. The HaCaT cells (8 × 10^3^) were exposed to 500 µg/mL concentration of FLPS and incubated. After every 24 h, the cells were washed three times with PBS, fixed with 3.7% formaldehyde, stained with DAPI, and examined under a fluorescence microscope (EVOS™ FLoid™ Cell Imaging Station, Life Technologies, Carlsbad, CA). Another set of washed cells was supplemented with fresh medium (without FLPS), incubated, and used to evaluate the retention and exclusion activity. Post-incubation, the cells were washed, fixed, and stained for microscopic examination.

#### Role of protein-corona in the NPs uptake

The influence of protein-corona in the NPs uptake was demonstrated using the fluorescent-labelled polystyrene NPs (FLPS) and Nile red-stained PENPs. The fluorescent FLPS and PENPs were treated with the human keratin solution to produce keratin-coronated NPs. Detailed Nile red staining procedure and keratin-corona preparation are presented in the Additional file 1. These coronated fluorescent NPs were exposed to the keratinocytes and incubated. After 30 and 60 min of exposure, the cells were washed and observed under a fluorescence microscope.

#### Mode of NPs internalization in cell

The NPs treated cells were fixed in glutaraldehyde (2%), harvested by scraping, and then embedded in resin for sectioning under an ultramicrotome (Leica ultra-cut UCT UC7, Austria) [99]. The sections were placed on a copper grid, stained with 4% uranyl acetate and 1% lead citrate, and observed under the HR-TEM (Technai, G2 20 Twin, FEI, USA). Here, we used spherical PSNPs for visual sorting and robust identification of particles under electron microscopy.

#### Lysosome labelling with neutral red

The keratinocytes exposed to FLPS were washed three times with PBS and immersed with the neutral red solution (2 mM) for 5 min. Then the cells were washed thrice with PBS, mounted, and observed under a confocal laser scanning microscope (CLSM) (Zeiss LSM 710, Carl Zeiss, Germany) [100].

#### Morphometry of NPs eliminated from the keratinocytes

After removing the medium containing the uninternalized PSNPs, the cells were washed three times and incubated with a fresh medium without PSNPs. After 24 h incubation, the cell-free medium was collected and centrifuged at 4000 rpm for 5 min. The pellet was dissolved in ultra-pure water, drop coated immediately onto a TEM grid, air-dried, and observed under HR-TEM. Similarly, the NPs incorporated in the sterile DMEM were also examined under HR-TEM to compare the morphology of the NPs [15]. Here, we used spherical PSNPs for visual sorting and robust identification of particles under electron microscopy.

### Assessment of cellular response against internalized NPs

#### Effect of single-dose on cell proliferation

To determine the effect of a single dose of lethal- and sub-lethal-concentrations of NPs on the cell proliferation, the HaCaT cells (3 × X 10^5^) were seeded in a 24-well plate and treated with a single dose of 50 and 100 µg /mL of NPs and 50 and 100 µM of H_2_O_2_ as a positive control for proliferation inhibition. After 48 h incubation, the NPs containing medium was removed, and the cells were washed with PBS and fresh medium to remove the particles attached to the cell surface and culture dish. Then the cells were harvested by trypsinization to estimate the viable cells using the trypan blue staining, wherein the dead cells stained in blue colour and viable cells remain unstained [101]. About 3 × 10^4^ cells (internalized with NPs) were seeded in triplicates into four individual culture plates and incubated for 24, 48, 72, and 96 h, respectively. Here, the culture medium containing the excreted NPs was withdrawn and replaced with fresh, NPs-free DMEM at every 24 h interval. After incubation, the cells were harvested, stned with 0.4% trypan blue, and counted in triplicates using a hemocytometer [102]. The relative difference of cell proliferation rate was calculated from the triplicate experiments using the following equation;

$${\text{Proliferation}}\,{\text{Index}}\,({\text{PI)}} = \frac{{\sum\nolimits_{1}^{i} {i \times \frac{{N_{i} }}{{2^{i} }}} }}{{\sum\nolimits_{1}^{i} {\frac{{N_{i} }}{{2^{i} }}} }}$$

where i is the generation number, and N_i_ is the number of events in generation i.

$${\text{Relative}}\;{\text{difference}} = \frac{{{\text{PI}}_{{\text{T}}} - {\text{PI}}_{{\text{C}}} }}{{\left( {\frac{{{\text{PI}}_{{\text{T}}} + {\text{PI}}_{{\text{C}}} }}{{2}}} \right)}}$$

where PI_T_ is the proliferation index of treated cells and PI_C_ is the proliferation index of control.

#### Senescence associated β-galactosidase assay

For assessing the cellular senescence, HaCaT cells (5X10^4^) were exposed to PENPs (10, 100 & 500 µg/mL), PSNPs (10, 100 & 500 µg/mL), NPs-1 (10, 50 & 100 µg/mL), NPs-2 (10, 50 & 100 µg/mL) and H_2_O_2_ (10, 50 & 100 µM) for 48 h. After 48 h of incubation, the medium (with NPs) was removed, cells were washed with PBS as described above, then the fresh, NPs-free medium was introduced and incubated. After 72 h, the cells were fixed using paraformaldehyde (3.7%) and stained for senescence-associated beta-galactosidase (SA-b-gal) using Senescence β-Galactosidase Staining Kit according to the manufacturer's instructions. Five phase-contrast images were captured in random locations using Carl Zeiss inverted microscope (Axio Vert.A1 FL, Carl Zeiss, Germany). The number of SA-β-gal positive cells (stained in blue) in respective concentrations was calculated and quantified using ImageJ 1.8.0 software (NIH) [44].

### Statistical analysis

All the treatment conditions were triplicated, and each experiment was repeated twice at different times, and all the data were presented as mean ± SME. For MTT data, a two-way analysis of variance (ANOVA) was carried out by considering the different concentrations of the NPs at specific treatment times as main factors. Tukey post hoc method was implemented for the pairwise multiple means comparison using OriginPro 2020b (Learning Edition, OriginLab Corp, Massachusetts, USA). The ROS data was also processed with two-way ANOVA with Tukey pairwise multiple means comparison for the treatment time of each treatment concentration in GraphPad Prism 8.4.3 (GraphPad Software, San Diego, CA, USA). Similar pairwise multiple means comparison and one-way ANOVA with Holm-Sidak multiple comparison analysis were done for SA β-gal positive cells and MDA activity, respectively. The statistical significance differences in the SOD and CAT activity between control and treatment groups were analysed by two-way ANOVA implementing Dunnett's multiple comparisons test using GraphPad Prism. The *p* < 0.05 was considered to indicate a statistically significant difference.

## Supplementary Information

**Additional file 1**. Supplementary information of NPs isolation, Fluorescent NPs preparation, MFI, Keratin-corona formation, digestion of NPs, plastic molecules release, and senescence activity.

## Data Availability

Not applicable.

## Abbreviations

CAT: Catalase
CLSM: Confocal laser scanning microscope
CPCP: Cosmetics and personal care products
DMEM: Dulbecco’s modified Eagle’s medium
FS: Face scrubs
FLPS: Fluorescently labelled polystyrene NPs
HR-TEM: High-resolution transmission electron microscopy
NPs-1: Nanoplastics isolated from FS-1
NPs-2: Nanoplastics isolated from FS-2
NPs/MPs: Nano-plastics/microplastics
PENPs: Polyethylene nano-plastics
PSNP: Polystyrene nano-plastics
ROS: Reactive oxygen species
SOD: Superoxide dismutase
