# Supplementary material for: Prospects on the nano-plastic particles internalization and induction of cellular response in human keratinocytes
Source: Part Fibre Toxicol. 2021 Sep 8;18:35. doi: 10.1186/s12989-021-00428-9 (PMC8424902; doi:10.1186/s12989-021-00428-9)
Supplement: Supplementary file 1 — Additional file 1. Supplementary information of NPs isolation, Fluorescent NPs preparation, MFI, Keratin-corona formation, digestion of NPs, plastic molecules release, and senescence activity. [file 12989_2021_428_MOESM1_ESM.docx]

**Prospects on the nano-plastic particles internalization and induction of cellular response in human keratinocytes**

**Ponnusamy Manogaran Gopinath^a^, Krishna Sundar Twayana^b^, Palaniyandi Ravanan*^b,c^, John Thomas^a^, Amitava Mukherjee^a^, David F Jenkins^d^ & Natarajan Chandrasekaran*^a^**

^a^Centre for Nanobiotechnology, Vellore Institute of Technology (VIT), Vellore – 632 014, Tamil Nadu, India

^b^Apoptosis and Cell Survival Research Lab, Department of Biosciences, School of Biosciences and Technology, VIT University, Vellore – 632 014, Tamil Nadu, India

^c^Department of Microbiology, School of Life Sciences, Central University of Tamil Nadu

Thiruvarur -610 104, Tamil Nadu, India

^d^Faculty of Science and Environment, Plymouth University, Plymouth PL4 8AA, UK

*** Corresponding authors**

**Dr. Natarajan Chandrasekaran**

Centre for Nanobiotechnology, Vellore Institute of Technology (VIT), Vellore, Tamil Nadu

Tel-: +91 416 2202624; fax: +91 416 2243092

E-mail address: [nchandrasekaran@vit.ac.in](mailto:nchandrasekaran@vit.ac.in), [nchandra40@hotmail.com](mailto:nchandra40@hotmail.com)

**Dr. P. Ravanan**

Department of Microbiology, School of Life Sciences, Central University of Tamil Nadu

Thiruvarur – 610 104, Tamil Nadu.

E-mail address: ravanan@cutn.ac.in

**Results**

**Isolation of MPs and NPs from FS samples**

FS-1 and FS-2 products contained around 27.62 ± 5.83 mg of plastic particles with the size ranging from 30 nm – 372 µm per gram of product and around 41.59 ± 7.47 mg of plastic particles with the size ranging from 100 nm – 225 µm per milliliter of product, respectively. In the four-step filtration, an average of 12.51 mg MPs that are > 25 µm size, 6. 88 mg MPs within 25 – 2.5 µm range, 4. 27 mg MPs between 2.5 -0.4 µm range, 2.94 mg NPs within 400 – 200 nm size and 2.15 mg NPs of <200 nm size were found in one gram of FS-1 product. Similarly, one mL of FS-2 contained 16.81 mg, 10.27 mg, 6.05 mg, 6.37 mg, and 4.44 mg of MPs/NPs, respectively. Because of the heterogeneousness of size and shape, a nonsignificant correlation between the mass and particle number was observed. As a result, the number of particles applied to the skin could differ in each application, however, particle mass may remain constant at every use. For example, the use of 400 mg FS-1 product per wash may allow nearly 10 mg of MPs and 1.5 mg of NPs to contact the skin, whereas 500 µl of FS-2 leads to exposure of around 20 mg of MPs and 2.2 mg of NPs onto the skin. We perceived from the preliminary studies that washing may remove up to 60 – 80 % of particles from the skin.

**Table S1. Dynamic light scattering size distribution and zeta potential analysis of NPs suspended in the distilled water, keratin solution (0.2%), and cell culture medium.**

| **DLS properties of Nanoplastics** | | **Dispersion medium** | | | |
| --- | --- | --- | --- | --- | --- |
|  |  | **Distilled water** | | **Keratin solution (0.2%)** | **Culture medium (DMEM)** |
|  |  | **<1 hr** | **24 hr** | **24 hr** | **24 hr** |
| **PENPs** | **Z- average (nm)**  **PDI**  **Zeta potential (mV)** | 321±71  2.938  -32±3.2 | 1409±520  9.106  -9±2.6 | 305±73  0.487  -56±6.0 | 333±19  0.534  -52±7.1 |
| **PSNPs** | **Z- average (nm)**  **PDI**  **Zeta potential (mV)** | 186±32  0.180  -38±6.1 | 179±39  0.201  -38±1.1 | 96±10  0.034  -43±5.3 | 156±18  0.143  -36±4.0 |
| **NPs-1** | **Z- average (nm)**  **PDI**  **Zeta potential (mV)** | 180±46  0.309  -42±1.7 | 192±25  0.311  -40±2.8 | 186±31  0.294  -46±2.0 | 354±11  0.468  -32±1.6 |
| **NPs-2** | **Z- average (nm)**  **PDI**  **Zeta potential (mV)** | 113±6.2  0.230  -50±1.3 | 120±7  0.242  -51±0.8 | 110±9.2  0.310  -51±1.7 | 376 ±7.7  0.501  -25±2.0 |

**Table S2. The mean MTT values of three independent experiments of the keratinocytes exposed to different NPs particles for 24, 48, 72, 96, 120, and 144 hrs.**

| **Sample** | **Time** | **Concentration** | | | | | |
| --- | --- | --- | --- | --- | --- | --- | --- |
|  |  | **0µg/ml** | **25µg/ml** | **50µg/ml** | **100µg/ml** | **250µg/ml** | **500µg/ml** |
| **PENPs** | **24 hrs** | 0.456 | 0.436 | 0.388 | 0.48 | 0.358 | 0.459 |
|  |  | 0.423 | 0.411 | 0.427 | 0.419 | 0.38 | 0.427 |
|  |  | 0.401 | 0.4 | 0.399 | 0.376 | 0.41 | 0.409 |
|  | **48 hrs** | 0.818 | 0.815 | 0.752 | 0.781 | 0.464 | 0.497 |
|  |  | 0.687 | 0.832 | 0.757 | 0.659 | 0.515 | 0.502 |
|  |  | 0.747 | 0.821 | 0.694 | 0.616 | 0.42 | 0.592 |
|  | **72 hrs** | 1.518 | 1.55 | 1.685 | 1.556 | 1.195 | 0.922 |
|  |  | 1.465 | 1.608 | 1.472 | 1.45 | 1.097 | 0.712 |
|  |  | 1.578 | 1.606 | 1.42 | 1.606 | 0.778 | 0.7125 |
|  | **96 hrs** | 2.22 | 2.829 | 3.406 | 3.112 | 2.54 | 1.127 |
|  |  | 2.674 | 2.448 | 2.649 | 2.968 | 1.752 | 1.139 |
|  |  | 2.499 | 2.717 | 3.193 | 3.092 | 1.826 | 0.999 |
|  | **120 hrs** | 3.468 | 3.323 | 3.557 | 3.301 | 2.969 | 1.091 |
|  |  | 3.252 | 3.552 | 2.983 | 2.858 | 2.729 | 1.299 |
|  |  | 3.693 | 3.239 | 3.056 | 3.267 | 3.012 | 1.111 |
|  | **144 hrs** | 5.38 | 6.449 | 4.537 | 4.193 | 4.901 | 2.197 |
|  |  | 5.448 | 4.835 | 4.203 | 4.337 | 4.783 | 1.877 |
|  |  | 5.056 | 5.773 | 4.157 | 4.951 | 5.215 | 2.001 |
| **PSNPs** | **Concentration** | | | | | | |
|  |  | **0µg/ml** | **25µg/ml** | **50µg/ml** | **100µg/ml** | **250µg/ml** | **500µg/ml** |
|  | **24 hrs** | 0.456 | 0.418 | 0.403 | 0.428 | 0.435 | 0.425 |
|  |  | 0.423 | 0.454 | 0.436 | 0.462 | 0.394 | 0.388 |
|  |  | 0.401 | 0.391 | 0.375 | 0.406 | 0.418 | 0.367 |
|  | **48 hrs** | 0.818 | 0.732 | 0.828 | 0.756 | 0.701 | 0.774 |
|  |  | 0.687 | 0.815 | 0.884 | 0.838 | 0.921 | 0.839 |
|  |  | 0.747 | 0.829 | 0.782 | 0.805 | 0.855 | 0.818 |
|  | **72 hrs** | 1.518 | 1.797 | 1.689 | 1.742 | 1.667 | 1.755 |
|  |  | 1.465 | 1.796 | 1.874 | 1.564 | 1.524 | 1.629 |
|  |  | 1.578 | 1.615 | 1.658 | 1.81 | 1.84 | 1.642 |
|  | **96 hrs** | 2.22 | 2.585 | 2.714 | 3.041 | 3.037 | 2.371 |
|  |  | 2.674 | 3.138 | 2.976 | 2.724 | 2.569 | 2.286 |
|  |  | 2.499 | 3.024 | 3.286 | 3.526 | 2.487 | 2.91 |
|  | **120 hrs** | 3.468 | 4.59 | 3.466 | 3.43 | 3.522 | 3.44 |
|  |  | 3.252 | 3.366 | 3.78 | 3.656 | 3.054 | 3.605 |
|  |  | 3.693 | 3.486 | 3.428 | 3.097 | 2.92 | 3.608 |
|  | **144 hrs** | 5.38 | 6.074 | 4.742 | 6.114 | 5.64 | 6.794 |
|  |  | 5.448 | 5.138 | 4.572 | 4.9 | 4.878 | 5.28 |
|  |  | 5.056 | 4.974 | 5.82 | 4.118 | 5.416 | 6.368 |
|  | | | | | | | |
| **NPs-1** | **Concentration** | | | | | | |
|  |  | **0µg/ml** | **25µg/ml** | **50µg/ml** | **100µg/ml** | **250µg/ml** | **500µg/ml** |
|  | **24 hrs** | 0.456 | 0.401 | 0.384 | 0.394 | 0.329 | 0.270 |
|  |  | 0.423 | 0.393 | 0.437 | 0.403 | 0.289 | 0.229 |
|  |  | 0.401 | 0.389 | 0.406 | 0.433 | 0.301 | 0.209 |
|  | **48 hrs** | 0.818 | 0.77 | 0.888 | 0.716 | 0.417 | 0.075 |
|  |  | 0.687 | 0.838 | 0.801 | 0.742 | 0.441 | 0.076 |
|  |  | 0.747 | 0.794 | 0.783 | 0.679 | 0.468 | 0.078 |
|  | **72 hrs** | 1.518 | 1.65 | 1.555 | 1.333 | 0.766 | 0.093 |
|  |  | 1.465 | 1.552 | 1.52 | 1.223 | 0.807 | 0.101 |
|  |  | 1.578 | 1.502 | 1.483 | 1.416 | 0.677 | 0.076 |
|  | **96 hrs** | 2.22 | 2.558 | 2.921 | 2.3 | 1.189 | 0.095 |
|  |  | 2.674 | 2.987 | 3.146 | 2.38 | 1.141 | 0.098 |
|  |  | 2.499 | 2.875 | 3.19 | 1.808 | 1.142 | 0.095 |
|  | **120 hrs** | 3.468 | 3.168 | 3.095 | 2.668 | 1.81 | 0.086 |
|  |  | 3.252 | 3.19 | 3.534 | 2.759 | 1.742 | 0.094 |
|  |  | 3.693 | 3.444 | 2.912 | 2.797 | 1.66 | 0.086 |
|  | **144 hrs** | 5.38 | 6.666 | 4.62 | 5.83 | 3.84 | 0.146 |
|  |  | 5.448 | 5.626 | 5.176 | 5.316 | 4.182 | 0.156 |
|  |  | 5.056 | 6.224 | 4.706 | 4.56 | 4.184 | 0.148 |
|  | | | | | | | |
| **NPs-2** | **Concentration** | | | | | | |
|  |  | **0µg/ml** | **5µg/ml** | **10µg/ml** | **50µg/ml** | **100µg/ml** | **250µg/ml** |
|  | **24 hrs** | 0.456 | 0.410 | 0.454 | 0.407 | 0.396 | 0.352 |
|  |  | 0.423 | 0.455 | 0.420 | 0.421 | 0.400 | 0.340 |
|  |  | 0.401 | 0.394 | 0.397 | 0.439 | 0.383 | 0.343 |
|  | **48 hrs** | 0.818 | 0.781 | 0.95 | 0.723 | 0.629 | 0.417 |
|  |  | 0.687 | 0.78 | 0.885 | 0.763 | 0.605 | 0.455 |
|  |  | 0.747 | 0.8 | 0.805 | 0.694 | 0.668 | 0.43 |
|  | **72 hrs** | 1.518 | 1.433 | 1.621 | 1.372 | 0.872 | 0.503 |
|  |  | 1.465 | 1.578 | 1.905 | 1.475 | 1.12 | 0.651 |
|  |  | 1.578 | 1.627 | 1.677 | 1.543 | 1.155 | 0.635 |
|  | **96 hrs** | 2.22 | 2.775 | 3.118 | 2.358 | 1.655 | 0.454 |
|  |  | 2.674 | 2.124 | 3.265 | 3.121 | 1.678 | 0.754 |
|  |  | 2.499 | 2.374 | 2.479 | 2.483 | 1.865 | 0.662 |
|  | **120 hrs** | 3.468 | 3.416 | 3.172 | 3.156 | 2.724 | 0.637 |
|  |  | 3.252 | 3.464 | 3.037 | 3.137 | 2.555 | 1.07 |
|  |  | 3.693 | 3.311 | 2.861 | 3.465 | 3.013 | 1.18 |
|  | **144 hrs** | 5.38 | 6.25 | 5.276 | 4.97 | 4.104 | 0.72 |
|  |  | 5.448 | 6.018 | 5.054 | 5.028 | 4.978 | 1.774 |
|  |  | 5.056 | 6.744 | 5.89 | 5.784 | 4.834 | 1.548 |
|  | | | | | | | |
| **H_2_O_2_** | **Concentration** | | | | | | |
|  |  | **0µM** | **25µM** | **50µM** | **100µM** | **250µM** | **500µM** |
|  | **24 hrs** | 0.456 | 0.39 | 0.362 | 0.394 | 0.318 | 0.303 |
|  |  | 0.423 | 0.371 | 0.38 | 0.39 | 0.329 | 0.31 |
|  |  | 0.401 | 0.387 | 0.339 | 0.36 | 0.335 | 0.294 |
|  | **48 hrs** | 0.818 | 0.788 | 0.7 | 0.812 | 0.519 | 0.463 |
|  |  | 0.687 | 0.726 | 0.801 | 0.735 | 0.599 | 0.393 |
|  |  | 0.747 | 0.717 | 0.689 | 0.729 | 0.557 | 0.393 |
|  | **72 hrs** | 1.518 | 1.407 | 1.585 | 0.851 | 0.637 | 0.572 |
|  |  | 1.465 | 1.424 | 1.382 | 0.861 | 0.612 | 0.567 |
|  |  | 1.578 | 1.577 | 1.329 | 0.978 | 0.698 | 0.552 |
|  | **96 hrs** | 2.22 | 2.142 | 2.671 | 1.587 | 1.021 | 0.517 |
|  |  | 2.674 | 2.59 | 2.352 | 1.599 | 0.75 | 0.704 |
|  |  | 2.499 | 2.688 | 2.39 | 1.946 | 0.697 | 0.618 |
|  | **120 hrs** | 3.468 | 3.259 | 2.5 | 1.942 | 1.007 | 0.598 |
|  |  | 3.252 | 3.242 | 2.451 | 2.338 | 1.194 | 0.576 |
|  |  | 3.693 | 2.451 | 3.073 | 2.238 | 1.236 | 0.458 |
|  | **144 hrs** | 5.38 | 4.794 | 5.468 | 4.754 | 2.926 | 0.756 |
|  |  | 5.448 | 3.874 | 5.3 | 4.434 | 2.466 | 0.684 |
|  |  | 5.056 | 4.778 | 4.748 | 4.684 | 2.938 | 0.592 |

**Figure S1. Intracellular ROS measured by DCFH2-DA staining of keratinocytes under fluorescence microscope.** (a) control, (b) PENPs (100 µg mL^-1^), (c) PSNPs (100 µg mL^-1^), (d) NPs-1 (100 µg mL^-1^), (e) NPs-2 (100 µg mL^-1^) and (f) H_2_O_2_ (100 µM). Scale bar - 125 µm.


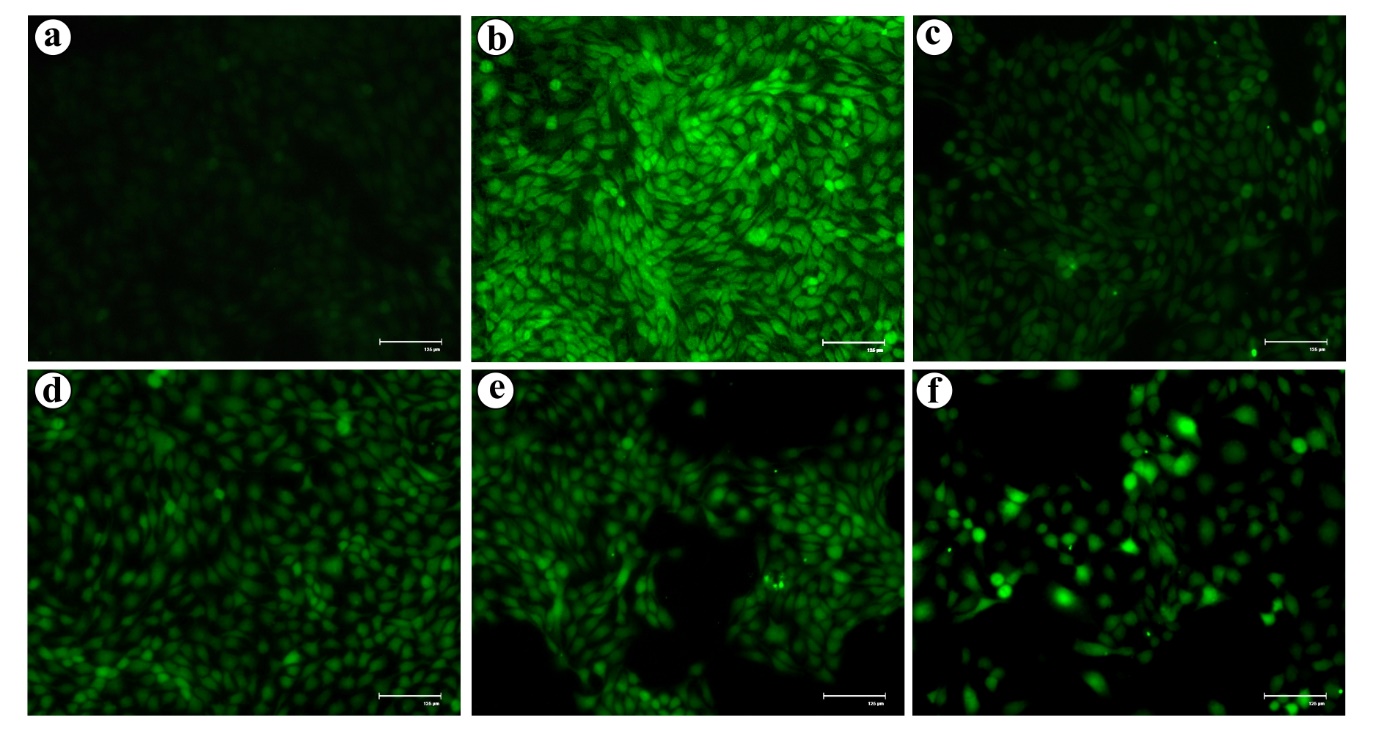


**Figure S2. Time-dependent internalization and exclusion of FLPS particles.** a) Phase-contrast, b) DAPI, c) GFP and d) superimposed images of HaCaT cells exposed to FLPS particles for 144 hrs. For fluorescent imaging, DAPI (λex: 357/44 nm; λem: 447/60 nm) and GFP (λex: 470/22 nm; λem: 510/42 nm) fluorescent light cubes were used. Scale bar: 20 μg.


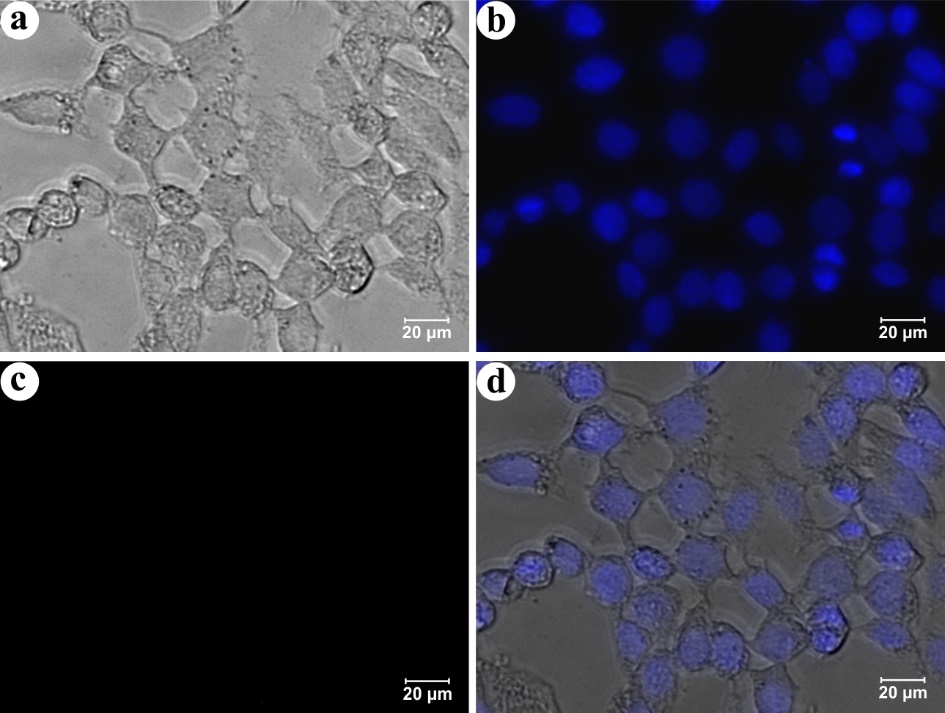


**Figure S3. Protein-corona formation with protein and smaller biomolecular aggregates on NPs surface. (**a) PENPs with 200 nm size protein corona, **(**b, c, and d) Corona mediated aggregates of 2 or 3 PSNPs, NPs-1 and NPs-2, respectively.


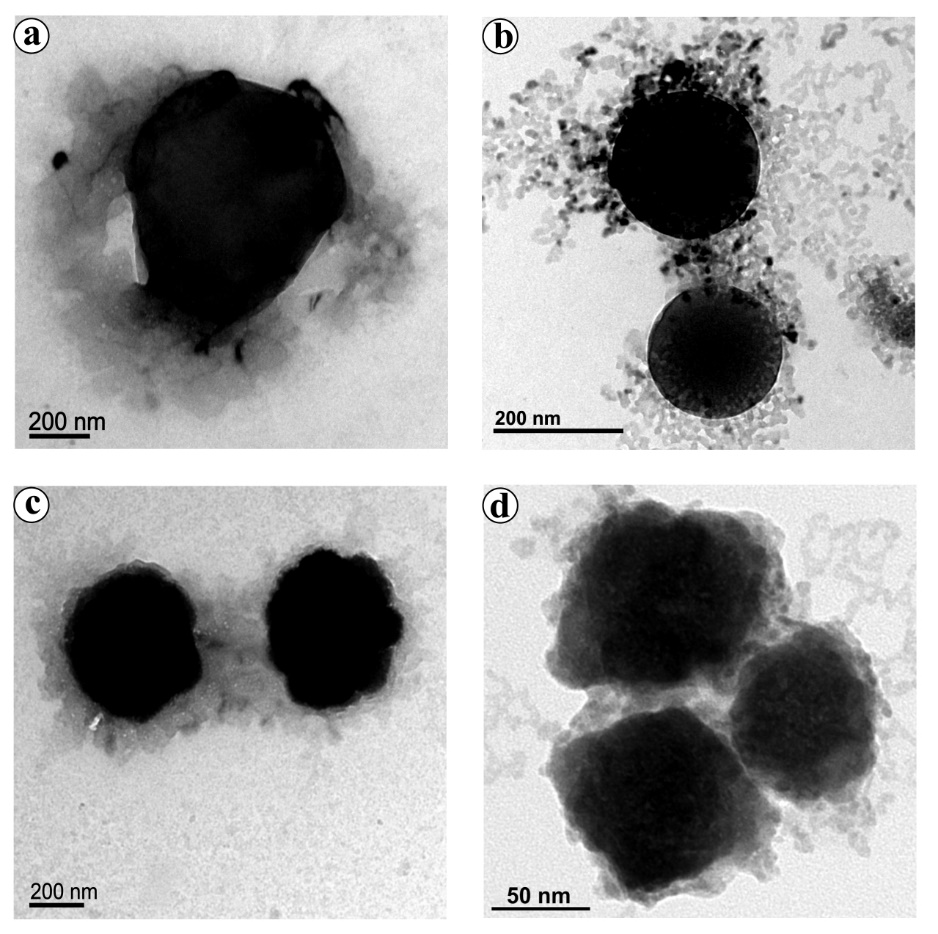


**Extraction of human hair keratin**

Human hair was washed with ethanol and incubated with chloroform/methanol (2: 1, v/v) mixture for 24 hrs to remove the external lipids. About 40 mg of the delipidated hair was mixed with 20 mL of a solution containing 25 mM Tris–HCl (pH 9.5), 8 M urea, and 5% 2-mercaptoethanol at 50 °C for 3 consecutive days. The mixture was filtered and centrifuged at 12000 rpm for 20 min at room temperature. The supernatant collected was dialyzed against 2 l of distilled water with 8—10 changes. The protein concentration in the final solution was determined by the Bradford protein assay using the Bio-Rad protein assay kit according to the manufacturer’s instructions. According to Nakamura et al. (2002), the final extract could be mainly composed of α-keratins and keratin-associated proteins. These α-keratins are resolved further into two subfamilies, such as Type I acidic and Type II neutral/basic keratins (Nakamura et al., 2002; Adav et al., 2018). Hence, the protein extract obtained from the human hair samples could contain both acidic and basic keratin as well as keratin-associated proteins. Therefore, the α-keratins and keratin-associated proteins might have formed keratin-corona on the NPs. Since this experiment aims to demonstrate the influence of protein corona on the rapid internalization of NPs, we have used crude keratin extract for protein-corona formation. It is noteworthy to mention that to maintain the reliability in corona formation on NPs and its effect on cells, we have used coronated-NPs derived from DMEM throughout the study.

**Figure. S4 HR-TEM micrograph of FLPS particles with thick keratin-corona**


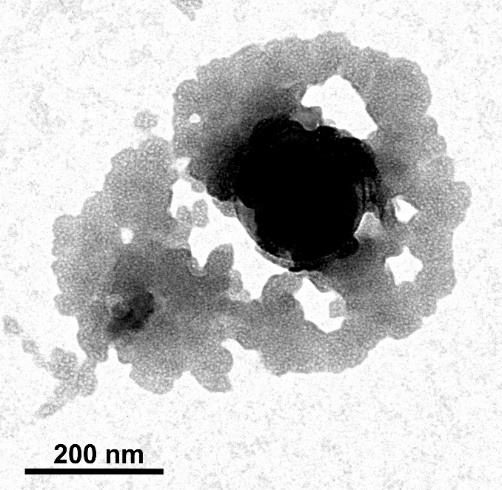


**Fluorescent-PENPs preparation and keratin-corona formation**

To demonstrate the influence of protein-corona in the rapid internalization of NPs, we have used fluorescently labeled polystyrene NPs (commercially procured) and the PENPs stained with the Nile red dye. For the preparation of fluorescent PENPs, we have followed a modified Nile red staining procedure to stain PENPs (Shim et al., 2016). Briefly, the dried PENPs (< 400nm) sample was dissolved in 5 mL of acetone containing Nile red dye (2µg/ mL) and mixed in a shaker incubator in dark conditions. After 5 min of mixing, the PENPs were separated by centrifugation, washed with acetone, and then dried under a vacuum dryer. Then the Nile red-stained PENPs was introduced into the human keratin solution (0.2%) and incubated for 1 hr for keratin-corona formation on PENPs. After incubation, the coronated-PENPs were separated by centrifugation (4000 rpm for 5 min) and used for the internalization studies. Here, the HaCaT cells (1x10^5^) were seeded in a culture dish and incubated to achieve confluence, and then the cells were treated with the keratin-coronated fluorescent-PENPs and -PSNPs. The fluorescent-NPs without keratin-corona were used as a control. At 30 and 60 min of exposure, the cells were harvested, washed, placed on a microscopic slide and covered with a coverslip, and then observed under a fluorescence microscope.

**Determination of mean fluorescence intensity of lysosomes and macropinosomes.**

The CLSM output file (.lsm file) for the lysosomal activity was processed using ImageJ software (<https://imagej.nih.gov/ij/download.html>). The red and green fluorescent channels were split for quantifying the mean fluorescence intensity (MFI) and area fluorescing in each channel. Using the freehand selection tool, the red fluorescence spots were drawn accurately, and measured the mean area, fluorescence, integrated density of the total fluorescing region (lysosomes) in the red channel. Similarly, the mean area, fluorescence, integrated density of the green fluorescing region (macropinosomes) was calculated from the green channel. Additionally, the yellow fluorescing regions (formation of macropinolysosomes) on the superimposed channels were also drawn and the measurements were obtained from the green channel. The background MFI of red and green channels was measured from the non-fluorescing regions on the image to avoid background interference (Shihan et al., 2021). Mean fluorescence intensities of the lysosomes, macropinosomes, and macropinolysosomes were calculated using the following formula, MFI = Integrated Density – (Mean fluorescing area x Mean background fluorescence). The percentage of lysosomal activity was calculated from the Red/Yellow fluorescence ratio and the percentage of macropinolysosomal activity was calculated from the Green/Yellow fluorescence ratio.

**Fig. S5. Mean fluorescence intensity of lysosomal activity.** Overlay images of red and green fluorescence of the control (a) and NPs treated cells (b) were obtained using CLSM. Red, green, and yellow fluorescence represent the lysosomes, macropinosomes, and macropinolysosomes, respectively. c) Mean fluorescence intensity derived from the integrated fluorescence density and d) percentage of lysosomal and macropinolysosomal activity.


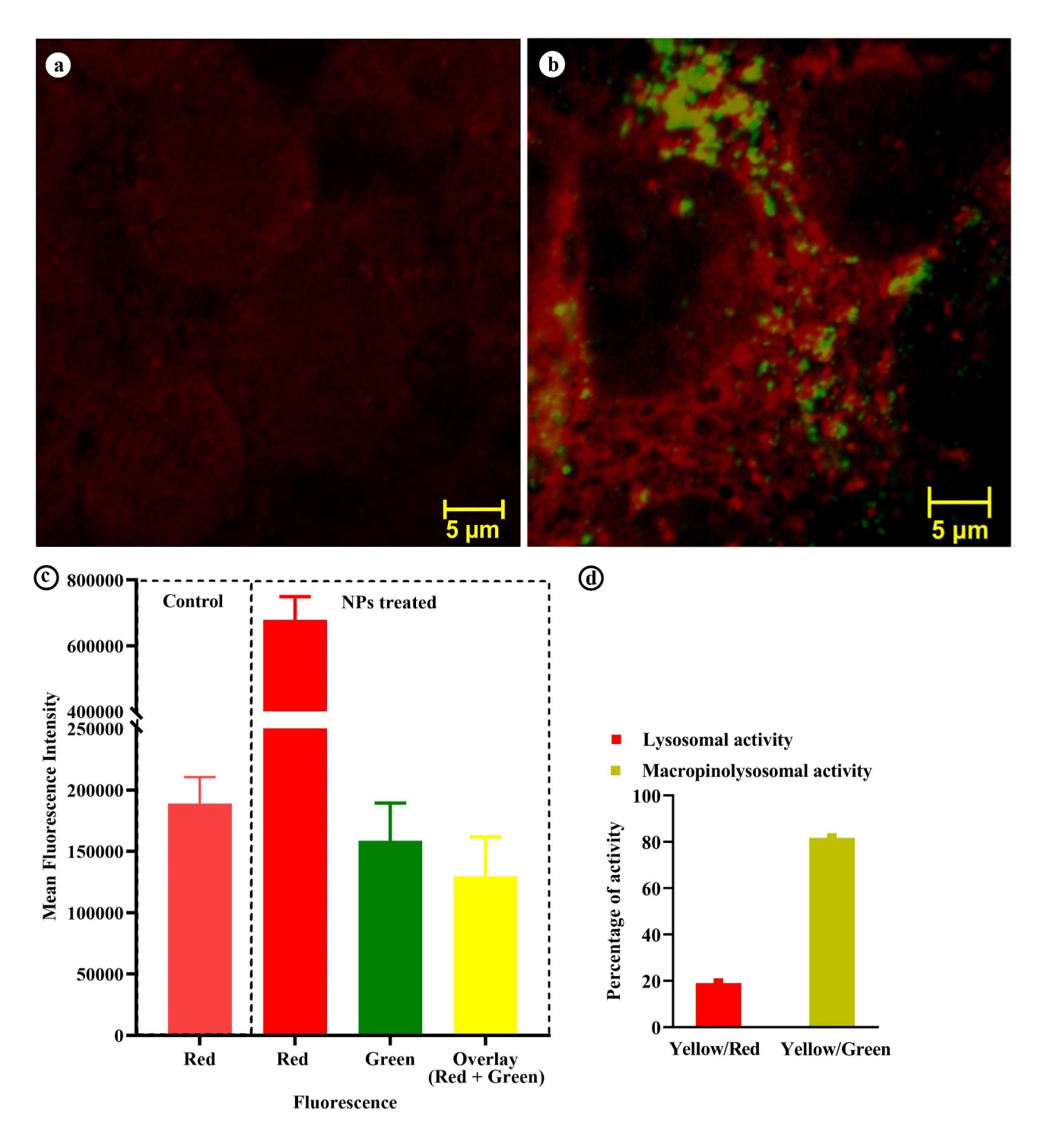


**Figure S6. Transmission electron micrographs showing the fate of the NPs post-macropinolysosomal process.** (a, b) coronated PSNPs without structural damage, (c-f) partly damaged, (g-l) disintegrated and (m-o) enlarged PSNPs.

**
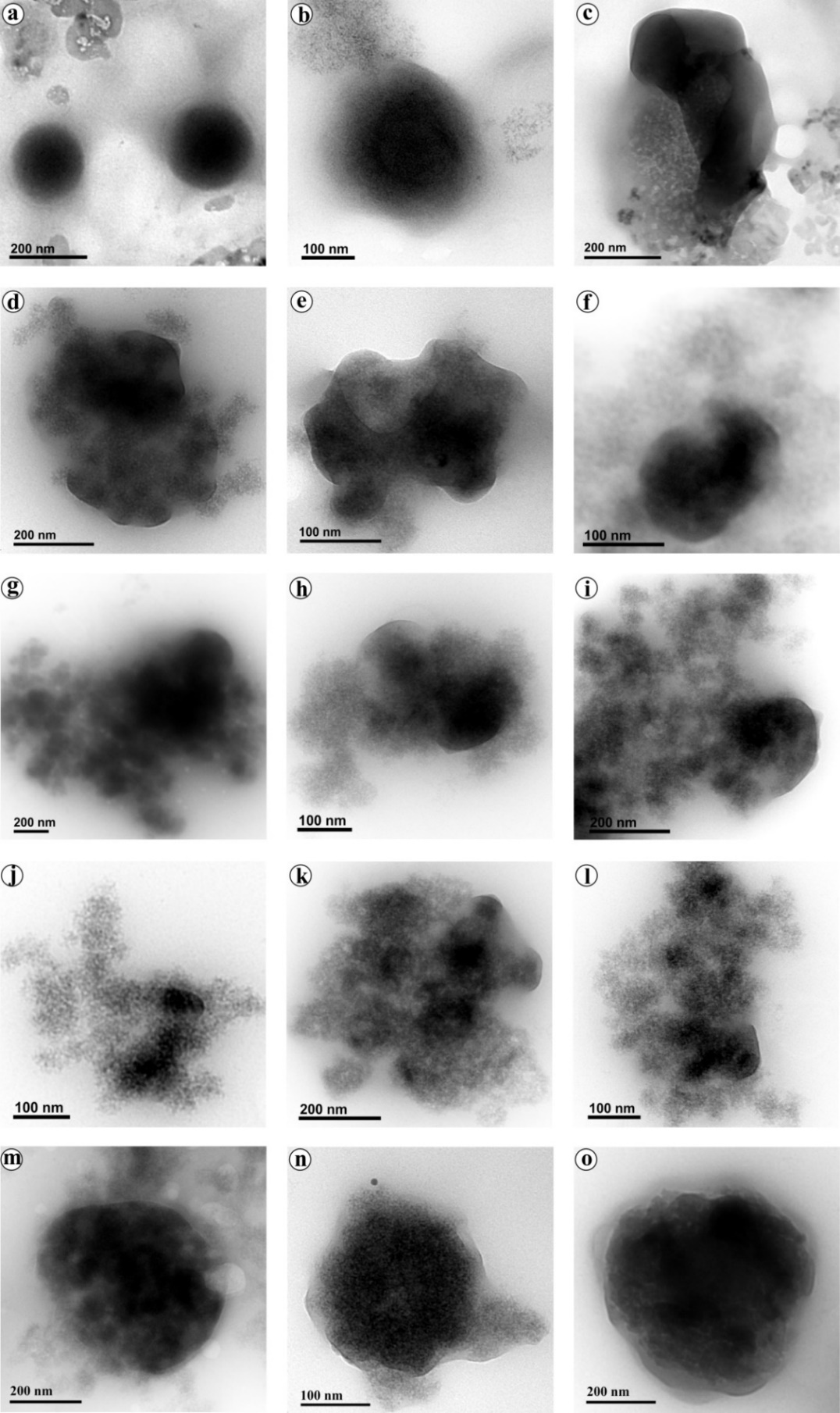
**

**Gas chromatography analysis of styrene molecules release**

The observed disintegration of PSNPs after lysosomal activity as described in this study has driven us to analyze the presence of styrene molecules in the cell-free supernatant collected from the PSNPs internalized cells. Herein, the coronated PSNPs without structural damage, partly damaged, disintegrated, and enlarged particles (Fig. S5) were separated from the withdrawn medium by centrifugation and the supernatant was subjected to the styrene molecules extraction according to Colin and Chakrabarti, 1986. The extracted styrene molecules were analyzed under gas chromatography along with the styrene standard (Sigma Aldrich, St. Louis, MO). It is noteworthy to mention that, the PSNPs incorporated in the culture medium was used as an experimental control. However, further optimization and standardization studies are needed to validate and quantify the molecular release with other NPs as well as its effect on the cells at molecular levels.

**Figure S7. Gas chromatography analysis of styrene molecules release from the macropinolysosomal activity in the PSNPs internalized cells.** Ten ng of styrene molecule (Sigma Aldrich, St. Louis, MO) was used as standard.


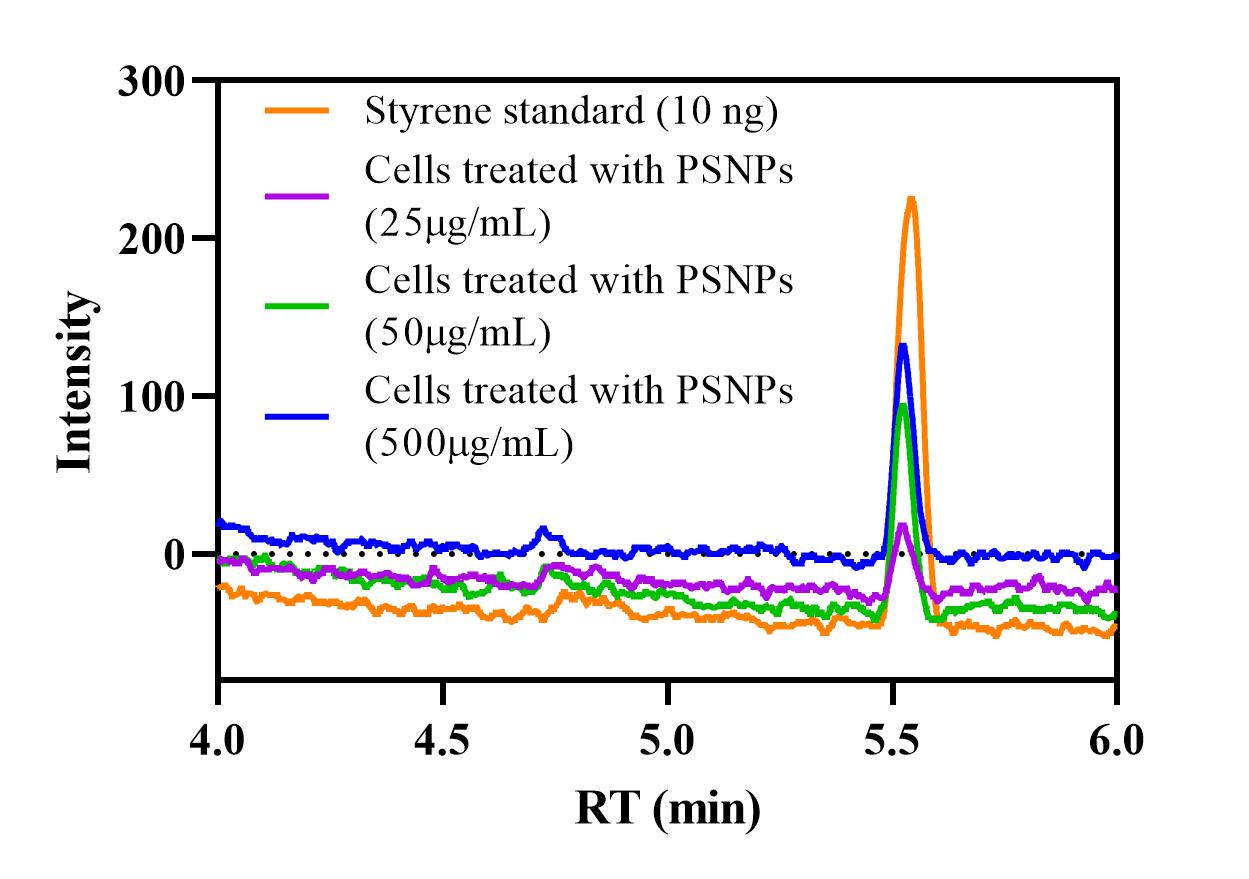


**Figure S8. NPs mediated induction of cellular senescence in the HaCaT cells.** (a-c) 10, 100 & 500 µg/mL of PENPs, (d-f) 10, 100 & 500 µg/mL of PSNPs, (g-i) 10, 50 & 100 µg/mL of NPs-1, ( j-l) 10, 50 & 100 µg/mL of NPs-2, (m) control and (n-o) 10 & 50 µM of H_2_O_2_, respectively. Scale bar - 60 µm.


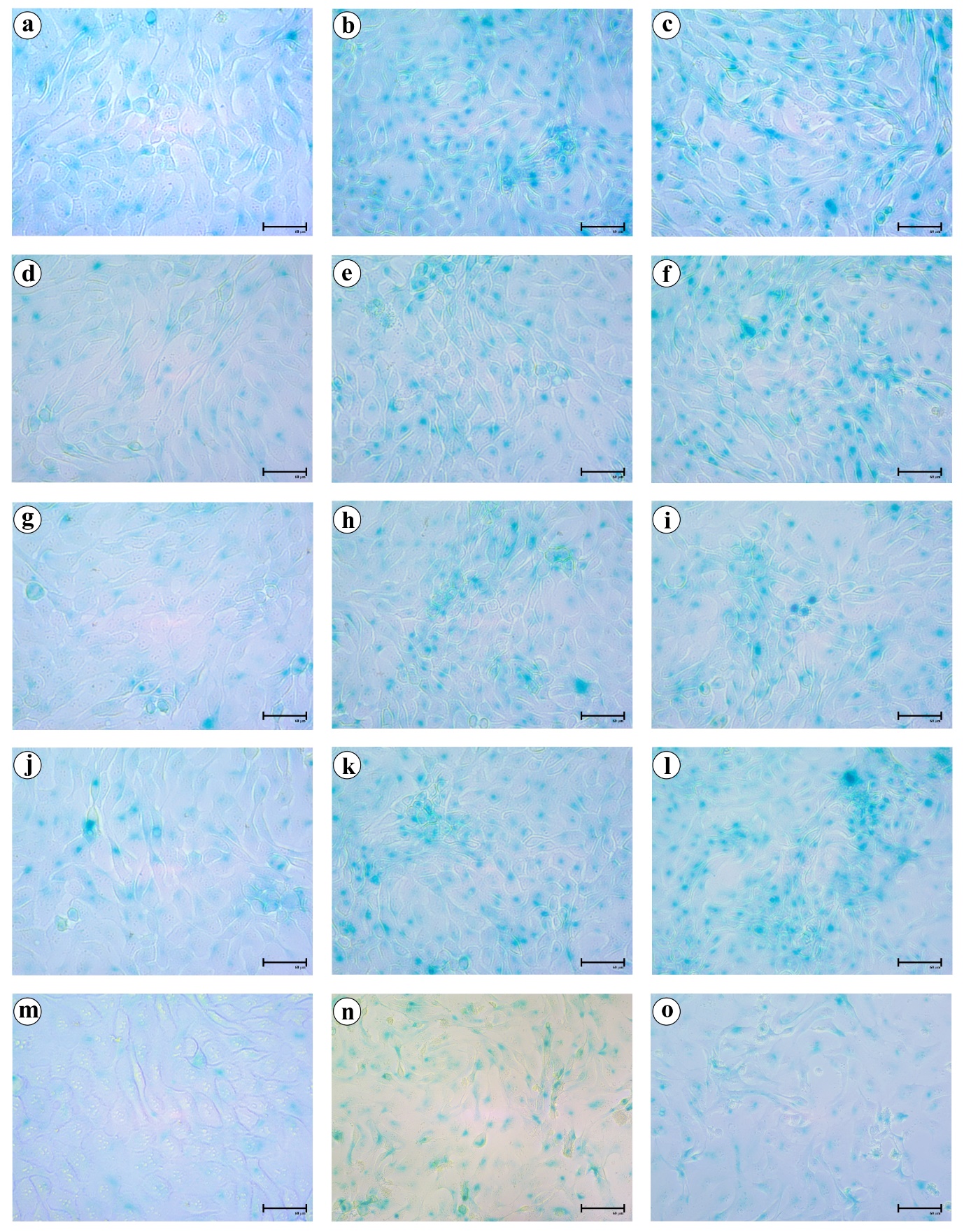


**Table S3. The percentage of SA-β-gal-positive cells in PENPs, PSNPs, NPs-1, NPs-2, and H_2_O_2_ treated cells and untreated cells**

|  | **Percentage of SA–β –gal–positive cells** | | |
| --- | --- | --- | --- |
| **Concentration** | **10 µg/mL** | **100 µg/mL** | **500 µg/mL** |
| **PENPs** | 56.2 ±2.9% | 75.4 ± 0.3% | 78.5 ± 2.6% |
| **PSNPs** | 50.5 ± 4.1% | 58.7 ± 2.0% | 85.2 ± 5.5% |
| **Concentration** | **10 µg/mL** | **50 µg/mL** | **100 µg/mL** |
| **NPs-1** | 29.2 ±1.8% | 57.1 ± 1.3% | 68.8 ± 6.5% |
| **NPs-2** | 51.7 ± 4.3% | 73.4 ± 2.8% | 87.6 ± 0.1% |
| **Concentration** | **10 µM** | **50 µM** | **100 µM** |
| **H_2_O_2_** | 71.8 ± 3.2% | 85.4 ± 2.0% | 96.0 ± 2.4 % |
| **Control** | 13.5 ± 2.2 % | | |

**Figure S9. HR-TEM micrograph of just filtered hot water (negative control)**


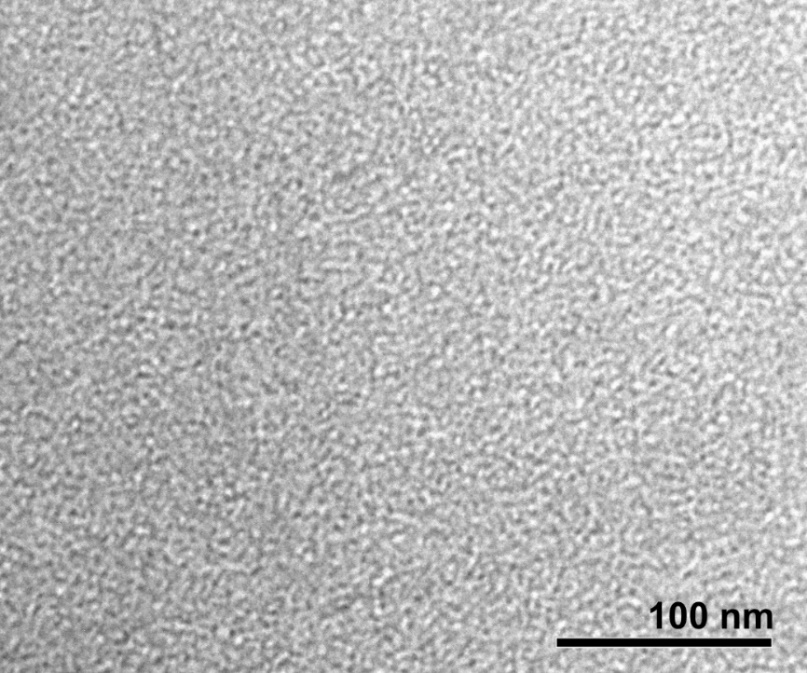


**Figure S10. FE-SEM micrograph of keratin-coated glass slides (negative control) with a) smooth surface and b) rough surface.**


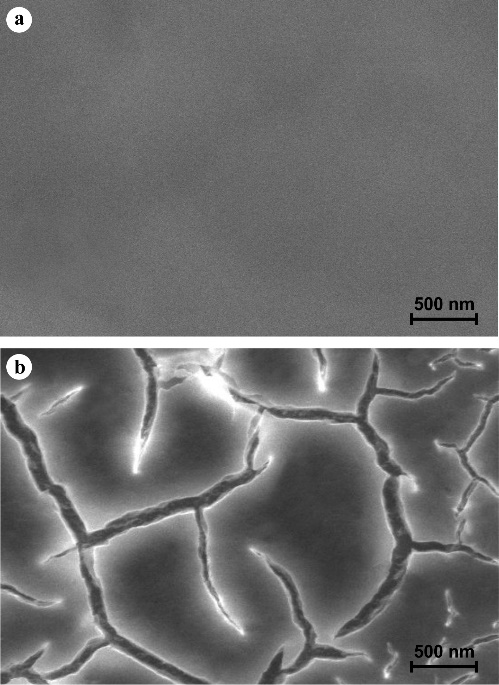


**References**

Adav, S. S., Subbaiaih, R. S., Kerk, S. K., Lee, A. Y., Lai, H. Y., Ng, K. W., ... & Schmidtchen, A. (2018). Studies on the proteome of human hair-Identification of histones and deamidated keratins. *Scientific reports*, *8*(1), 1-11.

Colin P, Sirois G, Chakrabarti S. Determination of styrene in biological samples by reversed-phase liquid chromatography. Journal of Chromatography. 1986;375 2:431-7.

Nakamura, A., Arimoto, M., Takeuchi, K., & Fujii, T. (2002). A rapid extraction procedure of human hair proteins and identification of phosphorylated species. *Biological and Pharmaceutical Bulletin*, *25*(5), 569-572.

Shihan, M. H., Novo, S. G., Le Marchand, S. J., Wang, Y., & Duncan, M. K. (2021). A simple method for quantitating confocal fluorescent images. Biochemistry and Biophysics Reports, 25, 100916.

Shim, W. J., Song, Y. K., Hong, S. H., & Jang, M. (2016). Identification and quantification of microplastics using Nile Red staining. *Marine pollution bulletin*, *113*(1-2), 469-476.
